# Supplementary material for: Measuring what matters: Context-specific indicators for assessing immunisation performance in Pacific Island Countries and Areas
Source: PLOS Glob Public Health. 2024 Jul 25;4(7):e0003068. doi: 10.1371/journal.pgph.0003068 (PMC11271932; doi:10.1371/journal.pgph.0003068)

**Measuring what matters: context-specific indicators for assessing immunisation performance in Pacific Island Countries and Areas**

# S1 Appendix: Expert elicitation instrument

Below is an extract of the instrument used for the expert elicitation. These questions were replicated for each category of indicators.


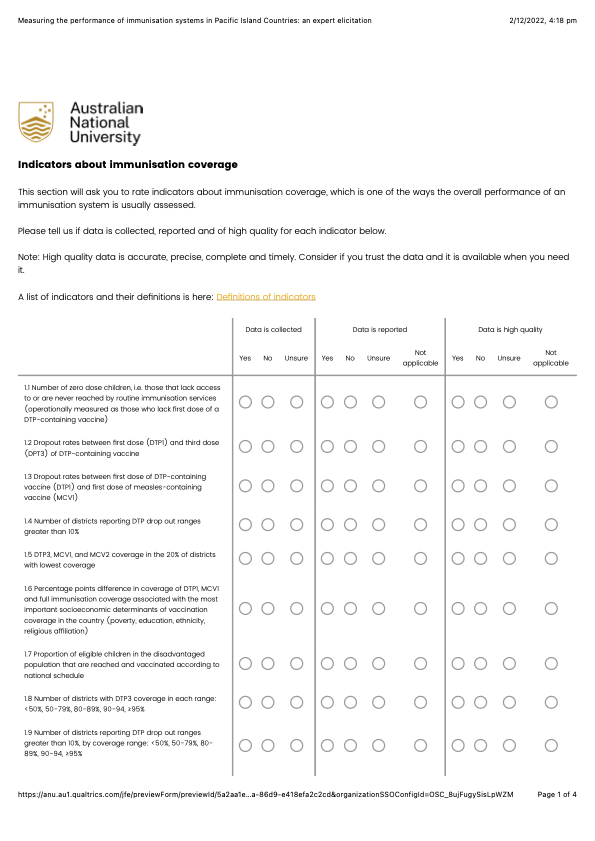

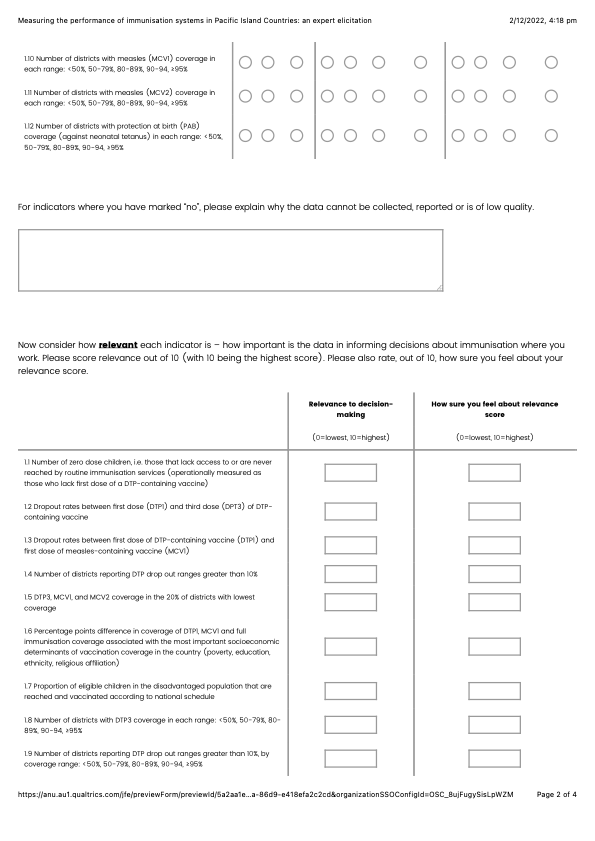

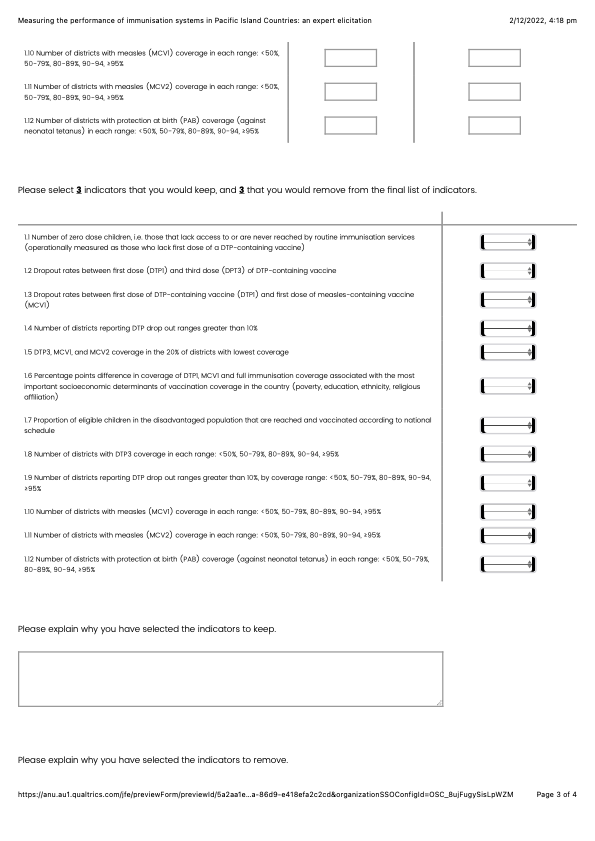


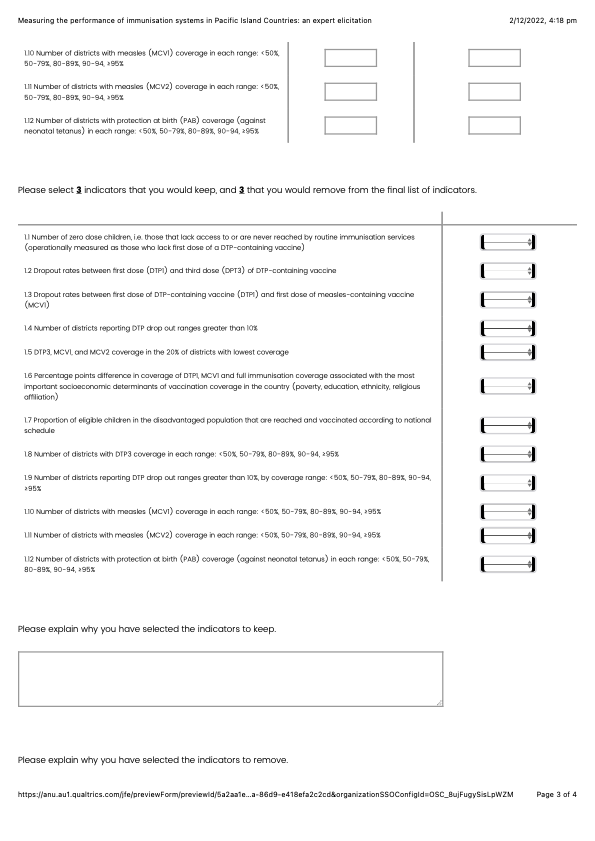

Supplement: S1 Appendix — (DOCX) [file pgph.0003068.s002.docx]
