# Supplementary material for: Measuring what matters: Context-specific indicators for assessing immunisation performance in Pacific Island Countries and Areas
Source: PLOS Glob Public Health. 2024 Jul 25;4(7):e0003068. doi: 10.1371/journal.pgph.0003068 (PMC11271932; doi:10.1371/journal.pgph.0003068)
Supplement: S2 Appendix — (DOCX) [file pgph.0003068.s003.docx]

# Measuring what matters: context-specific indicators for assessing immunisation performance in Pacific Island Countries and Areas

# S2 Appendix: Complete list of indicators included in the expert elicitation instrument

| **No.** | **Indicator** | **Type of indicator** | **Statistics / Numerator (for proportions)** | **Denominator (for proportions)** | **Notes** |
| --- | --- | --- | --- | --- | --- |
| Indicators about immunisation coverage | | | | | |
| 1.1 | Number of zero dose children, i.e. those that lack access to or are never reached by routine immunisation services (operationally measured as those who lack first dose of a DTP-containing vaccine) | Numeric (≥0) | The difference between the estimated number of surviving infants and the estimated number of children vaccinated with DTP1 | N/A | Requires data on coverage of the first dose of DTP vaccine |
| 1.2 | Dropout rates between first dose (DTP1) and third dose (DPT3) of DTP-containing vaccine | Numeric (≥0) | Difference in the coverage of DTP1 and DTP3 | N/A |  |
| 1.3 | Dropout rates between first dose (DTP1) and first dose of measles-containing vaccine (MCV1) | Numeric (≥0) | Difference in the coverage of DTP1 and MCV1 | N/A |  |
| 1.4 | Number of districts reporting DTP drop out ranges greater than 10% | Numeric (≥0) | Number of districts reporting a difference in the coverage of DTP1 and DPT3 greater than 10% | N/A | Requires coverage data by district |
| 1.5 | DTP3, MCV1, and MCV2 coverage in the 20% of districts with lowest coverage | Numeric (0–100%) | For each district with the lowest coverage, number of age-eligible children who received:   - DTP3 - MCV1 - MCV2 | For each district with the lowest coverage, number of children who are age-eligible to receive:   - DTP3 - MCV1 - MCV2 | Average coverage for each vaccine in the lowest-performing quintile. Requires analysis of district-level coverage. The group of worst performing districts may change from year to year. |
| 1.6 | Percentage points difference in coverage of DTP1, MCV1 and full immunisation coverage associated with the most important socioeconomic determinants of vaccination coverage in the country (poverty, education, ethnicity, religious affiliation) | Numeric (≥0) | For each vaccine group (DTP1, MCV1 and full immunisation), the difference in coverage between the highest and lowest grouping (e.g. percentile, quintile) by:   - Poverty - Education - Ethnicity - Religious affiliation - Other socioeconomic determinants of relevance | N/A | Data will be separate by vaccine type/group and by sociodemographic factor. Countries may choose to report data by other sociodemographic factors. |
| 1.7 | Proportion of eligible children in the disadvantaged population that are reached and vaccinated according to national schedule | Numeric (0–100%) | Number of eligible children in the disadvantaged population that are reached and vaccinated according to national schedule | Number of eligible children in the disadvantaged population |  |
| 1.8 | Number of districts with DTP3 coverage in each range: <50%, 50-79%, 80-89%, 90-94, ≥95% | Numeric (≥0) | Number of districts with DTP3 coverage between:   - <50% - 50–79% - 80–89% - 90–94% - ≥95% | N/A | Data on coverage of DTP3 by district is required |
| 1.9 | Number of districts reporting DTP drop out ranges greater than 10%, by coverage range: <50%, 50-79%, 80-89%, 90-94, ≥95% | Numeric (≥0) | Number of districts with DTP1–3 dropout ≥10% by range of DTP3 coverage between:   - <50% - 50–79% - 80–89% - 90–94% - ≥95% | N/A | Data on coverage of DTP1 and DTP3 by district is required to calculate dropout of coverage (DTP1–DTP3) by district |
| 1.10 | Number of districts with measles (MCV1) coverage in each range: <50%, 50-79%, 80-89%, 90-94, ≥95% | Numeric (≥0) | Number of districts with MCV1 coverage between:   - <50% - 50–79% - 80–89% - 90–94% - ≥95% | N/A | Data on coverage of MCV1 by district is required |
| 1.11 | Number of districts with measles (MCV2) coverage in each range: <50%, 50-79%, 80-89%, 90-94, ≥95% | Numeric (≥0) | Number of districts with MCV2 coverage between:   - <50% - 50–79% - 80–89% - 90–94% - ≥95% | N/A | Data on coverage of MCV2 by district is required |
| 1.12 | Number of districts with protection at birth (PAB) (against neonatal tetanus) coverage in each range: <50%, 50-79%, 80-89%, 90-94, ≥95% | Numeric (≥0) | Number of districts with PAB coverage between:   - <50% - 50–79% - 80–89% - 90–94% - ≥95% | N/A | A child is considered to have protection at birth against neonatal tetanus if the mother has received 2 doses of tetanus-toxoid vaccine in the last pregnancy or at least 3 doses in previous years.  Data on coverage of PAB by district is required. |
| Indicators about how insights are used | | | | | |
| 2.1 | Country uses quality data on under-vaccinated to inform plans at community, subnational and national levels | Categorical | Yes/no response | N/A |  |
| 2.2 | Proportion of stockpile applications that demonstrate use of evidence (e.g. disease surveillance data, root cause analysis, and coverage data) to support planning/targeting of outbreak response campaigns | Numeric (0–100%) | Number of stockpile applications that generate data that can be used to support outbreak response campaigns that have been used in planning or targeting the response | Number of stockpile applications that generate data that can be used to support outbreak response campaigns |  |
| 2.3 | Proportion of district health management committees (or equivalent at subnational level) that review immunisation performance as part of primary health care performance at least annually | Numeric (0–100%) | Number of district health management committees (or equivalent at subnational level) that review immunisation performance as part of primary health care performance at least annually | Total number of district health management committees (or equivalent at subnational level) |  |
| 2.4 | Commitment tracking and accountability frameworks used at country and subnational levels | Categorical | Yes/no response | N/A |  |
| Indicators about measuring data quality | | | | | |
| 3.1 | Proportion of live births registered | Numeric (0–100%) | Number of registered births | Number of live births |  |
| 3.2 | Proportion of districts with complete and timely reporting | Numeric (0–100%) | Number of districts submitting reports by the deadline set by the country with complete information | Total number of districts |  |
| 3.2a | Proportion of districts with complete and timely reporting from all health facilities | Numeric (0–100%) | Number of districts submitting reports by the deadline set by the country with complete information from all health facilities | Total number of districts |  |
| 3.3 | Proportion of districts reporting negative DTP1-DTP3 drop out | Numeric (0–100%) | Number of districts reporting negative DTP1-DTP3 coverage drop out | Total number of districts reporting data on DTP1 and DTP3 coverage |  |
| 3.4 | Proportion of districts with year-to-year variation of children vaccinated with DTP3 less than 15% | Numeric (0–100%) | Number of districts where the difference in DTP3 coverage between the current year and the previous year is <15% | Total number of districts reporting data on coverage of DTP3 in both years |  |
| 3.5 | Proportion of facility-level routine immunisation microplans with updated catchment area maps and strategy to reach them | Numeric (0–100%) | Number of health facilities that have updated their routine immunisation microplans with updated catchment area maps within the timeframe specified by the ministry of health and have a strategy to reach them | Number of health facilities that have routine immunisation microplans |  |
| 3.6 | Are the number of type-specific vaccine doses reported by age group (e.g. number of diphtheria cases by age group) based on recall, documentation, or both? | Categorical | Options include:   - Recall only - documentation only - both - neither | ­N/A |  |
| 3.7 | Does the private health sector deliver vaccines in your country and do you report it in your coverage? (Private health sector includes all organisations not owned or controlled by governments, including for-profit or not-for-profit, formal or informal, and domestic or foreign.) | Categorical | Yes/no response, but options may include:   - Delivers vaccine and included in coverage - Delivers vaccines, not included in coverage - Does not deliver vaccines | N/A |  |
| 3.8 | Proportion of districts reporting at least 90% on time during a one-year period for suspected cases for all priority vaccine-preventable diseases under nationwide surveillance, including reporting of zero cases | Numeric (0–100%) | Number of districts that have reported the number of all suspected priority VPD cases at least 90% of the time. | Total number of districts reporting data | The number of reports expected will depend on the frequency of reporting. E.g. if a country expects weekly reporting for a given disease then a district needs to report data ≥47 times by the deadline set by the country. |
| Indicators about data systems and processes | | | | | |
| 4.1 | Proportion of population with access to their personal immunisation records | Numeric (0–100%) | Number of people with access to personal immunisation records | Total population |  |
| 4.2 | Availability of sustainable and effective immunisation information system integrated within a robust national health information system | Categorical | Yes/no | N/A |  |
| 4.3 | Proportion of children with home-based immunisation records | Numeric (0–100%) | Number of children (of a specified age) with home-based immunisation records | Total number of children (or a specified age) | A home-based record is a health document used to record the history of health services received by an individual. It is kept in the household, in either paper (e.g. vaccination cards, child health books) or electronic format. |
| 3.4 | Linkage of home-based records with civil birth registration through immunisation services | Categorical | Yes/no | N/A | A home-based record is a health document used to record the history of health services received by an individual. It is kept in the household, in either paper (e.g. vaccination cards, child health books) or electronic format. |
| 4.5 | Proportion of districts with on-line access to health management information systems (HMIS) | Numeric (0–100%) | Number of districts with online access to HMIS | Number of districts |  |
| 4.6 | Proportion of districts having electronic vaccine and supply stock management system to monitor vaccine stock down to service delivery | Numeric (0–100%) | Number of districts having electronic vaccine and supply stock management system to monitor vaccine stock down to service delivery | Number of districts |  |
| 4.7 | Individual adverse event following immunisation (AEFI) case safety reports per million total population | Rate (n per 1,000,000) | Number of individual AEFI case safety reports | Total population |  |
| 4.8 | Is there a national system to monitor adverse events following immunisation (AEFIs)? | Categorical | Yes/no | N/A |  |
| 4.9 | Proportion of provinces/districts or other subnational units with at least one documented (with reporting form and/or line listed) individual serious AEFI case safety reports per million total population | Numeric (0–100%) | Number of provinces/districts reporting serious AEFIs at the rate of at least one individual serious AEFI case per million total population | Total number of provinces/districts |  |
| 4.10 | Proportion of districts reporting stock availability (vaccines and supplies) at a service delivery level | Numeric (0–100%) | Number of districts reporting stock availability (vaccines and supplies) at a service delivery level | Number of districts where vaccines are administered | Service delivery level refers to health facilities. |
| Indicators about vaccine-preventable disease surveillance systems | | | | | |
| 5.1 | Non-polio acute flaccid paralysis (AFP) rate (target >1/100,000 among <15 years population) in a 12-month period | Rate (n per 1,000,000) | Number of non-polio acute flaccid paralysis cases aged <15 years | Total population aged <15 years |  |
| 5.2 | Non-measles/non-rubella discard rate (target ≥2/100,000 population) | Rate (n per 1,000,000) | Number of suspected measles or rubella cases investigated and discarded as non-measles or non-rubella cases | Total population | Refers to the rate of suspected measles or rubella cases investigated and discarded as non-measles or non-rubella cases using laboratory testing and/or epidemiological linkage to another confirmed disease |
| 5.3 | Access to laboratory capacity to test for at least one bacterial vaccine-preventable disease (VPD) | Categorical | Yes/no | N/A |  |
| 5.4 | Proportion of polio, measles, meningococcal disease, yellow fever, cholera, and Ebola outbreaks with timely detection and response | Numeric (0–100%) | Number of known polio, measles, meningococcal disease, yellow fever, cholera and Ebola outbreaks with timely detection and outbreak response vaccination campaigns. | Number of known polio, measles, meningococcal disease, yellow fever, cholera and Ebola outbreaks with outbreak response vaccination campaigns | The acceptable time from onset of outbreak to campaign implementation should be defined for each disease, and consistent with WHO surveillance standards. |
| 5.5 | Annual number of laboratory-confirmed epidemic-prone vaccine-preventable disease outbreaks | Numeric (≥0) | Number of laboratory-confirmed epidemic-prone vaccine-preventable disease outbreaks in a given year | N/A |  |
| 5.6 | Does the country collect age and/or number of vaccine doses received for all cases of vaccine-preventable disease? | Categorical | Yes/no (response may also include ‘partial’) | N/A |  |

AEFI: Adverse event following immunisation; AFP: Acute flaccid paralysis; BCG: Bacille Calmette-Guérin vaccine; DTP: Diphtheria-tetanus-pertussis containing vaccines (includes combination vaccines that contain DTP and other antigens); MCV: Measles containing vaccine; OPV: Oral polio vaccine; Penta: Pentavalent vaccine; SIA: Supplementary immunisation activity; WHO: World Health Organization

A vaccine followed by a number indicators the dose number of that particular vaccine, for example:

- DTP1 = first dose of DTP vaccine
- DTP3 = third dose of DTP vaccine
- MCV = first dose of MCV
