# Supplementary material for: Measuring what matters: Context-specific indicators for assessing immunisation performance in Pacific Island Countries and Areas
Source: PLOS Glob Public Health. 2024 Jul 25;4(7):e0003068. doi: 10.1371/journal.pgph.0003068 (PMC11271932; doi:10.1371/journal.pgph.0003068)
Supplement: S3 Appendix — (DOCX) [file pgph.0003068.s004.docx]

**Measuring what matters: context-specific indicators for assessing immunisation performance in Pacific Island Countries and Areas**

# S3 Appendix: Statistical analysis of feasibility, relevance and preference scores, and ranking method

A detailed description of the method used to calculate feasibility, relevance and preference scores and method to rank indicates is provided here with a worked example. The data presented below are fictional and for illustrative purposes only. This example shows results obtained from a sample of six experts asked to rate five indicators.

Data collected via the expert elicitation rating rounds

The table below shows a fictional example of the raw data collected for a **single** indicator. This process was replicated for all indicators included in the rating (five indicators in this example).

*Table 3A: Raw data* collected for a single indicator from all experts included in the study*

| **Expert** | **Data is collected** | **Data is reported** | **Data is high quality** | **Relevance score** | **Confidence score** | **Keep** | **Remove** |
| --- | --- | --- | --- | --- | --- | --- | --- |
| A | Yes | Yes | Yes | 10 | 10 | X |  |
| B | Yes | Yes | Unsure | 8 | 2 | X |  |
| C | No | N/A | N/A | 5 | 7 |  | X |
| D | Unsure | Unsure | Unsure | 6 | 9 |  |  |
| E | Yes | No | Unsure | 4 | 5 |  |  |
| F | Yes | Yes | No | 9 | 7 | X |  |

*Note: these data are fictional and for illustrative purposes only

Calculation of feasibility scores

In our findings, we report the proportion of experts who selected “Yes” for each of the three feasibility items, i.e. that 1) data is collected, 2) data is reported, 3) data is of high quality. We defined “high quality” as data that is “accurate, precise, complete and timely”, and asked experts to consider whether they trusted the data and if it is available when they need it. We considered “unsure” or missing responses to be the equivalent of a “no” response for the purpose of analysis.

*Table 3B: Calculation of proportion of experts who selected “Yes” for each of the three feasibility items for a single indicator**

| **Expert** | **Data is collected** | **Data is reported** | **Data is high quality** |
| --- | --- | --- | --- |
| A | Yes | Yes | Yes |
| B | Yes | Yes | Unsure |
| C | No | N/A | N/A |
| D | Unsure | Unsure | Unsure |
| E | Yes | No | Unsure |
| F | Yes | Yes | No |
| Proportion | **4 / 6 = 67%** | **3 / 6 = 50%** | **1 / 6 = 17%** |

*Note: these data are fictional and for illustrative purposes only

We then calculated a feasibility score for each indicator. To do this, we assigned a score of “1” for each “Yes” response. We then calculated a feasibility score for each individual expert, and scaled it to a score out of 10. We then used the individual experts’ feasibility scores to calculate a mean feasibility score for the indicator.

*Table 3C: Calculation of feasibility score for a single indicator**

| **Expert** | **Data is collected** | **Data is reported** | **Data is high quality** | **Feasibility score, out of 3** | **Feasibility score, out of 10** |
| --- | --- | --- | --- | --- | --- |
|  | Based on expert’s response | Based on expert’s response | Based on expert’s response | Sum of responses to three items | Crude feasibility x 10 / 3 |
| A | 1 | 1 | 1 | 3 | 10.00 |
| B | 1 | 1 | 0 | 2 | 6.67 |
| C | 0 | 0 | 0 | 0 | 0.00 |
| D | 0 | 0 | 0 | 0 | 0.00 |
| E | 1 | 0 | 0 | 1 | 3.33 |
| F | 1 | 1 | 0 | 2 | 6.67 |
|  | 67% | 50% | 17% | – | **4.44** |

*Note: these data are fictional and for illustrative purposes only

Calculation of relevance scores

We calculated a crude and weighted mean relevance score for each indicator. To calculate the crude relevance score, we calculated the mean of the individual relevance scores provided by experts for the indicator.

We then calculated weighted relevance scores for each indicator, which were the crude relevance score weighted against the confidence score (i.e. how confident the expert felt about their relevance score). We used the confidence scores to create a weight for each individual expert. A weighted relevance score for each expert’s score for this specific indicator was calculated by multiplying their crude relevance score and the weight. The weights add up to the total number of participants included in the study (i.e. six in this worked example; 13 in the study). These means that weighted relevance scores for experts who expressed greater uncertainty in their score (i.e. had a lower confidence score) were lower than their crude relevance score, whereas those who expressed greater certainty had a higher weighted relevance score relative to their crude relevance score. In this way, experts who expressed greater certainty in their relevance score contributed proportionally more to the mean weighted relevance score.

The mean weighted relevance score for the indicator was the mean of all experts’ weighted relevance scores for the indicator. In the paper when we refer to relevance scores, we refer to the crude scores as “crude relevance scores”, and the weighted scores simply as “relevance scores”.

*Table 3D: Calculation of crude and weighted relevance scores for a single indicator**

| **Expert** | **Relevance score** | **Confidence score** | **Weight** | **Crude relevance score** | **Relevance score (i.e. weighted)** |
| --- | --- | --- | --- | --- | --- |
|  | Provided by expert | Provided by expert | Expert’s confidence score / Mean of all confidence scores | Based on relevance scores provided by expert | Expert’s relevance score x weight |
| A | 10 | 10 | 1.50 | 10 | 15.00 |
| B | 8 | 2 | 0.30 | 8 | 2.40 |
| C | 5 | 7 | 1.05 | 5 | 5.25 |
| D | 6 | 9 | 1.35 | 6 | 8.10 |
| E | 4 | 5 | 0.75 | 4 | 3.00 |
| F | 9 | 7 | 1.05 | 9 | 9.45 |
| Mean scores | – | – |  | **7.00** | **7.20** |

*Note: these data are fictional and for illustrative purposes only

Calculation of preference scores

Within each of the five indicator categories, we asked experts to select their top 25% indicators to “keep” and their bottom 25% indicators to “remove” in a single list of indicators to be used for immunisation performance monitoring across PICs. E.g. in the category for vaccination coverage, there were 12 indicators, so we asked experts to select three indicators that they would like to “keep” and three indicators they would like to “remove”. This meant that not all indicators would be selected to “keep” or “remove” by every expert. The purpose was to identify whether there was consensus among experts that, for at least some indicators, they were critical to measure across the Pacific, or consensus that at least some indicators were unimportant in the Pacific context and need not be monitored.

We calculated a preference score for each indicator based on experts’ preference to “keep” or “remove” the indicator. We summed the number of experts who selected the indicator to “keep” and those who selected the indicator to “remove”. The preference score was calculated as the number of experts who selected “keep” minus those who selected “remove” for the indicator.

*Table 3E: Calculation of a preference score for a single indicator**

| **Expert** | **Keep** | **Remove** |
| --- | --- | --- |
| A | X |  |
| B | X |  |
| C |  | X |
| D |  |  |
| E |  |  |
| F | X |  |
| All experts | 3 | 1 |

**Preference score for the indicator = 3 – 1 = 2**

*Note: these data are fictional and for illustrative purposes only

Ranking indicators to identify preferences for indicators

We followed the method above to calculate feasibility, relevance and preference scores for each indicator. To rank indicators, we first calculated a composite feasibility-relevance score for each indicator. This was done by calculating the mean of the feasibility and weighted relevance score for each of the five indicators included in this example. This composite score therefore equally weights the feasibility of data collection and reporting, and relevance of the indicator to decision-making.

*Table 3F: Calculation of composite feasibility-relevance score for each indicator**

| **Indicator** | **Mean feasibility score** | **Mean relevance score (weighted)** | **Mean feasibility-relevance score** |
| --- | --- | --- | --- |
|  | Calculated using method in Table 3C | Calculated using method in Table 3D | (Feasibility + Weighted Relevance) / 2 |
| Ind1 | 4.44 | 7.20 | 5.82 |
| Ind2 | 6.78 | 8.66 | 7.72 |
| Ind3 | 5.43 | 5.45 | 5.44 |
| Ind4 | 2.33 | 3.32 | 2.83 |
| Ind5 | 5.12 | 4.44 | 4.78 |

*Note: these data are fictional and for illustrative purposes only

We then ranked indicators based on the calculated preference and composite feasibility-relevance scores. We first ordered indicators based on the preference scores, and secondly on their composite feasibility-relevance scores. Higher scores were ranked higher (i.e. scores were ordered from highest to lowest).

*Table 3G: Method of ranking indicators based on preference and composite feasibility-relevance scores**

| **Indicator** | **Preference score** | **Mean composite feasibility-relevance score** |
| --- | --- | --- |
|  | Calculated using method in Table 3E | Calculated using method in Table 3F |
| Ind1 | 3 | 5.82 |
| Ind2 | 6 | 7.72 |
| Ind3 | 0 | 5.44 |
| Ind4 | -1 | 2.83 |
| Ind5 | 3 | 4.78 |

Ordered by: 1) preference score, 2) mean feasibility-relevance score

| **Indicator** | **Preference score** | **Mean composite feasibility-relevance score** | **Rank** |
| --- | --- | --- | --- |
| Ind2 | 6 | 7.72 | 1 |
| Ind1 | 3 | 5.82 | 2 |
| Ind5 | 3 | 4.78 | 3 |
| Ind3 | 0 | 5.44 | 4 |
| Ind4 | -1 | 2.83 | 5 |

*Note: these data are fictional and for illustrative purposes only

Results in Table 3 of the paper are reported in order of rank, with each indicator shown with its corresponding preference, mean composite feasibility-relevance, mean feasibility and mean relevance (weighted) scores.
