# Supplementary material for: Measuring what matters: Context-specific indicators for assessing immunisation performance in Pacific Island Countries and Areas
Source: PLOS Glob Public Health. 2024 Jul 25;4(7):e0003068. doi: 10.1371/journal.pgph.0003068 (PMC11271932; doi:10.1371/journal.pgph.0003068)
Supplement: S4 Appendix — (DOCX) [file pgph.0003068.s005.docx]

**Measuring what matters: context-specific indicators for assessing immunisation performance in Pacific Island Countries and Areas**

# S4 Appendix: Definitions of constructs and themes identified through discussions with expert elicitation participants

Construct 1: What is important to measure

- 1. Coverage
  2. Adverse events following immunisation (AEFI) and AEFI surveillance
  3. Data quality
  4. Surveillance
  5. Others – e.g. Vaccine supply, program planning

Construct 2: Why should an indicator be measured

- 1. Informs decision making about immunisation
  2. Priority disease
  3. Global and/or regional importance
  4. Provides information encompassing many outcomes/activities
  5. Feasible to collect, report and interpret

Construct 3: Why shouldn’t an indicator be measured

- 1. Burden of data collection and reporting
  2. Data is not used
  3. Not a priority
  4. Poor data quality
  5. Infrastructure limitations
  6. Poor wording of indicators

Construct 4: What factors explain discrepancies in indicator ratings

- 1. Diversity in health systems and priorities
  2. Infrastructure differences and limitations
  3. Population size
  4. Priorities, experience and influence of different actors
  5. other indicators preferred

Construct 5: Characteristics of indicator sets

- 1. Provides overview of immunisation
  2. Reduces redundancy
  3. Fewer/manageable number of indicators

Construct 6: Pacific Island Countries and Areas (PICs) context-specific factors

- 1. Small populations
  2. Population movement
  3. High coverage
  4. Issues with subnational level divisions
  5. Small/negligible private sector

**Definitions**

**1. What is important to measure: Includes discussion of which outcomes the expert thinks is important to measure for immunisation performance, and why it is more (or less) important than measuring other outcomes. This may also include reasons why certain aspects of these outcomes should be measured (that are specific to the outcome, rather than more general reasons that would be categorised under themes 2 and 3) and why they are more informative than measuring other outcomes. Also includes discussion of how these outcomes are best measured, and any challenges with measuring them.**

- 1. Coverage: Includes discussion of which coverage indicators are most important to measure. Includes discussion of coverage of individual vaccines, zero-dose or complete vaccination, coverage of vaccines across the lifespan, coverage of new vaccines or those of emerging importance (e.g. COVID-19), and dropout of vaccination coverage. Also note when certain indicators are less important to measure e.g. we don’t measure zero-dose because we have high coverage of childhood vaccination and it is not worth the resources. Includes discussion of coverage by sociodemographic factors and why these indicators are considered important/not important.
  2. AEFI and AEFI surveillance: Includes discussion that AEFI and AEFI surveillance are important. Also include discussion about what aspects of AEFIs and surveillance systems are important to measure, and which indicators provide useful information (and why). Includes discussion of socio-behavioural factors affecting AEFI reporting and the context that makes it challenging for AEFI surveillance systems to be effective.
  3. Data quality: Includes any discussion about data quality being an important concept to measure and challenges with measuring data quality.
  4. Surveillance: Includes discussion of what aspects of surveillance are important to measure, and the relationship with immunisation.
  5. Other: Includes discussion of other outcomes that experts identified as being important to measure, including human resource, vaccine supply and logistics, program planning (microplanning) and others. Also includes other data that were considered by experts to be important to have, e.g. population denominator data, data on information system performance.

1. **Why should an indicator be measured: These are reasons identified by experts to explain why the outcomes identified above are important to measure, and reasons for their preferences for which indicators should be measured.**
   1. Informs decision making about immunisation: Participant states that the data informs decision making about immunisation and is associated with a data use action. E.g. the data is important to understand immunisation performance, for planning and/or responding to outbreaks, for planning immunisation services, to identify at risk populations and target services, etc.
   2. Priority disease: The disease targeted by the vaccine is one of national importance, a priority for prevention and for outbreak control and management, and is a disease that is critical to control.
   3. Global and/or regional important: The indicator or disease is a global priority and/or that it is a critical indicator for disease prevention globally or regionally that must be reported.
   4. Provides information encompassing multiple outcomes/activities: The indicator provides an overarching view of the outcome, or where an indicator provides information on multiple activities that must be completed before the indicator is achieved (e.g. system for AE surveillance is established). Also includes indicators that are useful for/relevant to other health outcomes (e.g. birth registration relevant for population denominator used for calculation of other statistics).
   5. Feasible to collect, report and interpret: The indicator is preferred because the data is easy to collect, report and interpret, and therefore easy to use.
2. **Why shouldn’t an indicator be measured: These are reasons identified by experts to explain why it is not preferable to measure these outcomes or indicators.**
   1. Burden of data collection and reporting: Data for the indicator is difficult to collect, analyse and/or report. This may be because the indicator is complex and has many components (e.g. reporting coverage by different geographical or demographic factors), the data is available but difficult to analyse and report, and the data is not currently collected and would be difficult or burdensome to collect. This also includes discussion related to the burden of data collection and reporting in the context of limited resources especially human resources.
   2. Data is not used: The information provided by the indicator is not used because it doesn’t provide insights that would influence decisions about immunisation. E.g. the issue may not be a priority or a problem, or is not relevant to the current program or decisions being deliberated.
   3. Not a priority: The indicator or the outcome being measured is not a priority for the immunisation program or for decision making about immunisation. Reasons for this may include that it is not a current focus for the program, or that it is not a problem (e.g. coverage for a specific vaccine is high already so coverage of that vaccine is not a priority issue), or that other aspects of the program/other outcomes are more important.
   4. Issues with data quality: Data for the indicator are known or suspected to be poor. The participant may discuss this in the context of data being too difficult to obtain, or that the data can be obtained but it will be poor quality and therefore not useful to decision makers.
   5. Poor wording of indicators: The indicator is difficult to understand, measure and/or interpret because it is too complex, poor defined or without a standard agreed definition. E.g. the term “disadvantaged population” may be different in different countries and contexts. The wording of indicators may also be complex and difficult to understand, or use difficult or uncommon terminology, which may lead to confusion over what is being measured and how to measure it.
   6. Health system and infrastructure limitations: This relates to comments about the health system and health infrastructure in the Pacific that affects what can or can’t be measured. Includes discussion about limited laboratory capacity and absence of various committees for managing immunisation programs, and constraints of the existing infrastructure, especially the data systems and digital infrastructure, which may limit whether the data for an indicator can be collected.
3. **What factors explain discrepancies in indicator ratings: These are factors identified by experts that explain why some indicators are preferred in some settings but not others. They vary by the context, but also by the experience of the expert and what they believe is a priority.**
   1. Diversity in health systems and priorities: These are factors related to the existing health, immunisation systems or information systems that may affect whether it is feasible to collect data. Related discussions may pertain to the limitations of the current data systems or what data is able to be collected/reported in the country, or to the digital maturity / digital infrastructure in the setting. It also includes variations in the priorities of the country, based on what the desired health and system outcomes are. (e.g. adverse event surveillance systems).
   2. Infrastructure differences and limitations: These are factors related to variations in the existing data systems and infrastructure.
   3. Population size: These are factors related to variations in population size across PICs, and how this affects what can be measured and what the priorities for immunisation system monitoring might be.
   4. Priorities, experiences and influence of different actors: These include variations in the priorities and influence of different immunisation actors, including differences in priorities between global, regional and national levels, donor organisation vs recipient country, and differences across different roles (e.g. government employee, clinician and program manager). This includes discussions related to different in preferences based on who is accountable for the performance of the indicator. It can also include differences in what is perceived to be important based on their professional experiences and role(s).
   5. Other indicators preferred: This includes discussions where experts express that an indicator provides useful information, but another similar or related indicator is perceived to be even more useful, relevant or easier to collect.
4. **Characteristics of indicator sets: These are characteristics of sets of indicators that experts identify as being desirable.**
   1. Provides overview of immunisation: The set should provide an overview of the current status of the immunisation system and its performance. This includes discussion related to the perception that indicators that provide greater breadth of information rather than depth is preferable.
   2. Reduces redundancy: This is discussion related to experts’ preference for indicators in a set to be included to reduce redundancy.
   3. Fewer/manageable number of indicators: This is discussion related to experts’ preference for indicator sets to have a smaller number of indicators and the reasons for this. This includes having a manageable number of indicators to track that is feasible and easier for decision makers to absorb and use in their decision making.
   4. Frequency of reporting: This is discussion related to how frequently experts believed it was feasible and/or desirable to report immunisation data for the purposes of monitoring national performance.
5. **PICs context-specific factors: These are contextual factors identified through the discussions related to the PICs context, that are common across interviews (but are notably distinct from other parts of the world).**
   1. Small populations: This is discussion related to the small size of the populations in all PICs, and how this affects which indicators are more relevant and useful.
   2. Population movement: This is discussion related to the highly mobile populations in PICs and the implications for collecting and interpreting immunisation data, especially at the subnational level.
   3. High coverage: This is discussion related to general comments that routine childhood immunisation coverage in PICs is generally high and the implications for preferences for which indicators should be tracked. This is particularly relevant to discussion regarding indicators that may be prioritised in other settings or at the global level, but may not be as relevant in PICs.
   4. Issues with subnational level divisions: This relates to comments made about the relevance of reporting at subnational levels in PICs where there are small yet highly mobile populations. It includes comments about what subnational data is more useful, and preferences to track high level indicators at the national level rather than at subnational levels. It also includes discussion about tracking indicators at the district level, and how generally experts did not think that tracking district-level indicators was useful or a good use of resources.
   5. Small/negligible private sector: This relates to discussions about how the private sector is small or negligible for immunisation services in PICs, and the implications for whether data on the private sector needs to be captured. It also includes any comments about how the private sector may have
