# Supplementary material for: Measuring what matters: Context-specific indicators for assessing immunisation performance in Pacific Island Countries and Areas
Source: PLOS Glob Public Health. 2024 Jul 25;4(7):e0003068. doi: 10.1371/journal.pgph.0003068 (PMC11271932; doi:10.1371/journal.pgph.0003068)
Supplement: S5 Appendix — (DOCX) [file pgph.0003068.s006.docx]

# Measuring what matters: context-specific indicators for assessing immunisation performance in Pacific Island Countries and Areas

# S5 Appendix: Feasibility scores – proportion of experts who believed data for the indicator was collected, reported and of high quality, and mean feasibility scores, by indicator

| **Category** | **Indicator** | **% collected** | **% reported** | **% high quality** | **Mean feasibility score** |
| --- | --- | --- | --- | --- | --- |
| Immunisation coverage | Number of zero dose children, i.e. those that lack access to or are never reached by routine immunisation services (operationally measured as those who lack first dose of a DTP-containing vaccine) | 76.9% | 76.9% | 30.8% | 6.15 |
|  | Dropout rates between first dose (DTP1) and third dose (DPT3) of DTP-containing vaccine | 84.6% | 69.2% | 23.1% | 5.90 |
|  | Dropout rates between first dose (DTP1) and first dose of measles-containing vaccine (MCV1) | 76.9% | 46.2% | 23.1% | 4.87 |
|  | Number of districts reporting DTP drop out ranges greater than 10% | 53.8% | 38.5% | 25.0% | 3.85 |
|  | DTP3, MCV1, and MCV2 coverage in the 20% of districts with lowest coverage | 61.5% | 53.8% | 23.1% | 4.62 |
|  | Percentage points difference in coverage of DTP1, MCV1 and full immunisation coverage associated with the most important socioeconomic determinants of vaccination coverage in the country (poverty, education, ethnicity, religious affiliation) | 23.1% | 25.0% | 16.7% | 2.05 |
|  | Proportion of eligible children in the disadvantaged population that are reached and vaccinated according to national schedule | 15.4% | 15.4% | 7.7% | 1.28 |
|  | Number of districts with DTP3 coverage in each range: <50%, 50-79%, 80-89%, 90-94, ≥95% | 69.2% | 61.5% | 46.2% | 5.90 |
|  | Number of districts reporting DTP drop out ranges greater than 10%, by coverage range: <50%, 50-79%, 80-89%, 90-94, ≥95% | 61.5% | 53.8% | 23.1% | 4.62 |
|  | Number of districts with measles (MCV1) coverage in each range: <50%, 50-79%, 80-89%, 90-94, ≥95% | 84.6% | 69.2% | 41.7% | 6.41 |
|  | Number of districts with measles (MCV2) coverage in each range: <50%, 50-79%, 80-89%, 90-94, ≥95% | 69.2% | 61.5% | 30.8% | 5.38 |
|  | Number of districts with protection at birth (PAB) (against neonatal tetanus) coverage in each range: <50%, 50-79%, 80-89%, 90-94, ≥95% | 69.2% | 46.2% | 15.4% | 4.36 |
| Use of insights | Country uses quality data on under-vaccinated to inform plans at community, subnational and national levels | 69.2% | 58.3% | 25.0% | 4.87 |
|  | Proportion of stockpile applications that demonstrate use of evidence (e.g. disease surveillance data, root cause analysis, and coverage data) to support planning/targeting of outbreak response campaigns | 61.5% | 46.2% | 23.1% | 4.36 |
|  | Proportion of district health management committees (or equivalent at subnational level) that review immunisation performance as part of primary health care performance at least annually | 23.1% | 23.1% | 7.7% | 1.79 |
|  | Commitment tracking and accountability frameworks used at country and subnational levels | 7.7% | 8.3% | 7.7% | 0.77 |
| Data quality | Proportion of live births registered | 92.3% | 84.6% | 38.5% | 7.18 |
|  | Proportion of districts with complete and timely reporting | 69.2% | 76.9% | 23.1% | 5.64 |
|  | Proportion of districts with complete and timely reporting from all health facilities | 84.6% | 69.2% | 30.8% | 6.15 |
|  | Proportion of districts reporting negative DTP1-DTP3 drop out | 61.5% | 30.8% | 15.4% | 3.59 |
|  | Proportion of districts with year-to-year variation of children vaccinated with DTP3 less than 15% | 30.8% | 15.4% | 7.7% | 1.79 |
|  | Proportion of facility-level routine immunisation microplans with updated catchment area maps and strategy to reach them | 30.8% | 23.1% | 7.7% | 2.05 |
|  | Are the number of type-specific vaccine doses reported by age group (e.g. number of diphtheria cases by age group) based on recall, documentation, or both? | 69.2% | 66.7% | 23.1% | 5.13 |
|  | Does the private health sector deliver vaccines in your country and do you report it in your coverage? | 53.8% | 50.0% | 8.3% | 3.59 |
|  | Proportion of districts reporting at least 90% on time during a one-year period for suspected cases for all priority vaccine-preventable diseases under nationwide surveillance, including reporting of zero cases | 61.5% | 53.8% | 15.4% | 4.36 |
| Data systems and processes | Proportion of population with access to their personal immunisation records | 7.7% | 7.7% | 7.7% | 0.77 |
|  | Availability of sustainable and effective immunisation information system integrated within a robust national health information system | 46.2% | 46.2% | 23.1% | 3.85 |
|  | Proportion of children with home-based immunisation records | 30.8% | 23.1% | 15.4% | 2.31 |
|  | Linkage of home-based records with civil birth registration through immunisation services | 30.8% | 30.8% | 15.4% | 2.56 |
|  | Proportion of districts with on-line access to health management information systems (HMIS) | 38.5% | 33.3% | 16.7% | 2.82 |
|  | Proportion of districts having electronic vaccine and supply stock management system to monitor vaccine stock down to service delivery | 23.1% | 23.1% | 15.4% | 2.05 |
|  | Individual adverse event following immunisation (AEFI) case safety reports per million total population | 38.5% | 38.5% | 23.1% | 3.33 |
|  | Is there a national system to monitor adverse events following immunisation (AEFIs)? | 84.6% | 46.2% | 7.7% | 4.62 |
|  | Proportion of provinces/districts or other subnational units with at least one documented (with reporting form and/or line listed) individual serious AEFI case safety reports per million total population | 15.4% | 15.4% | 0.0% | 1.03 |
|  | Proportion of districts reporting stock availability (vaccines and supplies) at a service delivery level | 61.5% | 46.2% | 25.0% | 4.36 |
| Vaccine-preventable disease surveillance systems | Non-polio acute flaccid paralysis (AFP) rate (target >1/100,000 among <15 years population) in a 12-month period | 53.8% | 46.2% | 30.8% | 4.36 |
|  | Non-measles/non-rubella discard rate (target ≥2/100,000 population) | 46.2% | 50.0% | 25.0% | 3.85 |
|  | Access to laboratory capacity to test for at least one bacterial vaccine-preventable disease (VPD) | 53.8% | 45.5% | 27.3% | 3.85 |
|  | Proportion of polio, measles, meningococcal disease, yellow fever, cholera, and Ebola outbreaks with timely detection and response | 53.8% | 38.5% | 23.1% | 3.85 |
|  | Annual number of laboratory-confirmed epidemic-prone vaccine-preventable disease outbreaks | 46.2% | 38.5% | 33.3% | 3.85 |
|  | Does the country collect age and/or number of vaccine doses received for all cases of vaccine-preventable disease? | 46.2% | 38.5% | 7.7% | 3.08 |

AEFI: Adverse events following immunisation; AFP: Acute flaccid paralysis; DTP: diphtheria-tetanus-pertussis; HMIS: health management information system; MCV: measles-containing vaccine; PAB: protection at birth; VPD: vaccine-preventable disease
