# Supplementary material for: Measuring what matters: Context-specific indicators for assessing immunisation performance in Pacific Island Countries and Areas
Source: PLOS Glob Public Health. 2024 Jul 25;4(7):e0003068. doi: 10.1371/journal.pgph.0003068 (PMC11271932; doi:10.1371/journal.pgph.0003068)
Supplement: S6 Appendix — (DOCX) [file pgph.0003068.s007.docx]

**Measuring what matters: context-specific indicators for assessing immunisation performance in Pacific Island Countries and Areas**

# S6 Appendix: Mean crude and weighted relevance scores and confidence scores, by indicator

| **Category** | **Indicator** | **Mean crude relevance score (SD)** | **Mean confidence score (SD)** | **Mean weighted relevance score (SD)** |
| --- | --- | --- | --- | --- |
| Immunisation coverage | Number of zero dose children, i.e. those that lack access to or are never reached by routine immunisation services (operationally measured as those who lack first dose of a DTP-containing vaccine) | 8.17 (2.98) | 8.17 (3.24) | 8.47 (4.92) |
|  | Dropout rates between first dose (DTP1) and third dose (DPT3) of DTP-containing vaccine | 8.33 (2.84) | 8.50 (2.39) | 8.36 (4.08) |
|  | Dropout rates between first dose (DTP1) and first dose of measles-containing vaccine (MCV1) | 8.67 (1.56) | 9.17 (1.34) | 8.76 (2.38) |
|  | Number of districts reporting DTP drop out ranges greater than 10% | 7.75 (2.73) | 8.50 (1.51) | 7.78 (3.50) |
|  | DTP3, MCV1, and MCV2 coverage in the 20% of districts with lowest coverage | 7.08 (2.88) | 8.17 (2.59) | 7.61 (4.38) |
|  | Percentage points difference in coverage of DTP1, MCV1 and full immunisation coverage associated with the most important socioeconomic determinants of vaccination coverage in the country (poverty, education, ethnicity, religious affiliation) | 7.08 (3.53) | 8.67 (2.43) | 7.00 (4.44) |
|  | Proportion of eligible children in the disadvantaged population that are reached and vaccinated according to national schedule | 7.08 (3.92) | 8.83 (2.33) | 7.33 (4.78) |
|  | Number of districts with DTP3 coverage in each range: <50%, 50-79%, 80-89%, 90-94, ≥95% | 7.42 (3.34) | 8.08 (3.06) | 8.03 (5.13) |
|  | Number of districts reporting DTP drop out ranges greater than 10%, by coverage range: <50%, 50-79%, 80-89%, 90-94, ≥95% | 7.25 (2.80) | 7.83 (3.04) | 7.96 (4.74) |
|  | Number of districts with measles (MCV1) coverage in each range: <50%, 50-79%, 80-89%, 90-94, ≥95% | 8.08 (2.39) | 9.00 (1.54) | 8.29 (3.26) |
|  | Number of districts with measles (MCV2) coverage in each range: <50%, 50-79%, 80-89%, 90-94, ≥95% | 8.67 (1.88) | 9.00 (1.71) | 8.98 (3.02) |
|  | Number of districts with protection at birth (PAB) (against neonatal tetanus) coverage in each range: <50%, 50-79%, 80-89%, 90-94, ≥95% | 6.83 (3.16) | 7.92 (2.75) | 7.30 (4.98) |
| Use of insights | Country uses quality data on under-vaccinated to inform plans at community, subnational and national levels | 9.00 (1.73) | 9.54 (0.88) | 9.05 (2.17) |
|  | Proportion of stockpile applications that demonstrate use of evidence (e.g. disease surveillance data, root cause analysis, and coverage data) to support planning/targeting of outbreak response campaigns | 7.92 (2.33) | 8.54 (2.15) | 8.28 (3.61) |
|  | Proportion of district health management committees (or equivalent at subnational level) that review immunisation performance as part of primary health care performance at least annually | 6.69 (2.69) | 8.31 (2.46) | 6.76 (3.63) |
|  | Commitment tracking and accountability frameworks used at country and subnational levels | 6.69 (2.66) | 7.92 (2.47) | 7.11 (4.14) |
| Data quality | Proportion of live births registered | 8.58 (1.78) | 8.67 (1.88) | 8.92 (3.23) |
|  | Proportion of districts with complete and timely reporting | 7.67 (3.06) | 8.75 (1.55) | 7.79 (3.80) |
|  | Proportion of districts with complete and timely reporting from all health facilities | 7.42 (3.12) | 8.42 (2.02) | 7.70 (4.21) |
|  | Proportion of districts reporting negative DTP1-DTP3 drop out | 6.75 (2.70) | 7.58 (1.98) | 6.80 (3.80) |
|  | Proportion of districts with year-to-year variation of children vaccinated with DTP3 less than 15% | 5.83 (2.69) | 6.92 (2.64) | 6.12 (4.60) |
|  | Proportion of facility-level routine immunisation microplans with updated catchment area maps and strategy to reach them | 7.25 (3.79) | 9.17 (1.53) | 7.34 (4.35) |
|  | Are the number of type-specific vaccine doses reported by age group (e.g. number of diphtheria cases by age group) based on recall, documentation, or both? | 7.82 (1.89) | 8.00 (2.10) | 8.24 (3.63) |
|  | Does the private health sector deliver vaccines in your country and do you report it in your coverage? | 4.92 (3.34) | 7.83 (2.29) | 4.97 (3.92) |
|  | Proportion of districts reporting at least 90% on time during a one-year period for suspected cases for all priority vaccine-preventable diseases under nationwide surveillance, including reporting of zero cases | 7.92 (2.11) | 8.42 (1.56) | 8.12 (3.14) |
| Data systems and processes | Proportion of population with access to their personal immunisation records | 6.85 (3.02) | 7.77 (2.59) | 7.26 (4.44) |
|  | Availability of sustainable and effective immunisation information system integrated within a robust national health information system | 8.62 (2.18) | 8.69 (1.93) | 9.01 (3.45) |
|  | Proportion of children with home-based immunisation records | 6.39 (3.48) | 7.77 (3.09) | 7.14 (4.59) |
|  | Linkage of home-based records with civil birth registration through immunisation services | 7.39 (2.84) | 8.77 (1.48) | 7.45 (3.43) |
|  | Proportion of districts with on-line access to health management information systems (HMIS) | 7.69 (2.75) | 8.08 (2.84) | 8.54 (3.79) |
|  | Proportion of districts having electronic vaccine and supply stock management system to monitor vaccine stock down to service delivery | 7.92 (2.96) | 8.23 (2.86) | 8.84 (3.94) |
|  | Individual adverse event following immunisation (AEFI) case safety reports per million total population | 7.15 (2.38) | 8.23 (2.09) | 7.47 (3.67) |
|  | Is there a national system to monitor adverse events following immunisation (AEFIs)? | 8.08 (2.87) | 8.31 (2.87) | 8.97 (3.85) |
|  | Proportion of provinces/districts or other subnational units with at least one documented (with reporting form and/or line listed) individual serious AEFI case safety reports per million total population | 6.42 (2.88) | 7.83 (2.33) | 6.69 (4.09) |
|  | Proportion of districts reporting stock availability (vaccines and supplies) at a service delivery level | 8.54 (1.85) | 8.92 (1.66) | 8.80 (2.79) |
| Vaccine preventable disease surveillance systems | Non-polio acute flaccid paralysis (AFP) rate (target >1/100,000 among <15 years population) in a 12-month period | 7.75 (2.63) | 7.83 (3.22) | 8.36 (4.95) |
|  | Non-measles/non-rubella discard rate (target ≥2/100,000 population) | 7.17 (3.04) | 7.58 (3.23) | 7.77 (5.28) |
|  | Access to laboratory capacity to test for at least one bacterial vaccine-preventable disease (VPD) | 7.69 (1.80) | 7.69 (2.84) | 8.19 (4.04) |
|  | Proportion of polio, measles, meningococcal disease, yellow fever, cholera, and Ebola outbreaks with timely detection and response | 8.58 (1.68) | 8.50 (1.62) | 8.84 (3.01) |
|  | Annual number of laboratory-confirmed epidemic-prone vaccine-preventable disease outbreaks | 8.46 (1.56) | 8.69 (1.49) | 8.70 (2.79) |
|  | Does the country collect age and/or number of vaccine doses received for all cases of vaccine-preventable disease? | 7.75 (2.09) | 8.25 (1.96) | 8.09 (3.56) |

AEFI: Adverse events following immunisation; AFP: Acute flaccid paralysis; DTP: diphtheria-tetanus-pertussis; HMIS: health management information system; MCV: measles-containing vaccine; PAB: protection at birth; SD: standard deviation; VPD: vaccine-preventable disease
