# Supplementary material for: Measuring what matters: Context-specific indicators for assessing immunisation performance in Pacific Island Countries and Areas
Source: PLOS Glob Public Health. 2024 Jul 25;4(7):e0003068. doi: 10.1371/journal.pgph.0003068 (PMC11271932; doi:10.1371/journal.pgph.0003068)
Supplement: S7 Appendix — (DOCX) [file pgph.0003068.s008.docx]

**Measuring what matters: context-specific indicators for assessing immunisation performance in Pacific Island Countries and Areas**

# S7 Appendix: Ten highest ranked indicators, by differing rating criteria and ranking method (results of sensitivity analysis)

| **Rank** | **Indicator ID** | **Category** | **Definition** | **Preference score** | **Mean feasibility-relevance score** | **Mean weighted relevance score** | **Mean feasibility score** |
| --- | --- | --- | --- | --- | --- | --- | --- |
| **Alternative ranking method 1: Ordered by 1) preference score, 2) weighted relevance score, 3) feasibility score** | | | | | | | |
| 1 | 2.1 | Use of insights | Country uses quality data on under-vaccinated to inform plans at community, subnational and national levels | 10 | 6.96 | 9.05 | 4.87 |
| 2 | 4.2 | Data systems and processes | Availability of sustainable and effective immunisation information system integrated within a robust national health information system | 6 | 6.43 | 9.01 | 3.85 |
| 3 | 1.11 | Immunisation coverage | Number of districts with measles (MCV2) coverage in each range: <50%, 50-79%, 80-89%, 90-94, ≥95% | 6 | 7.18 | 8.98 | 5.38 |
| 4 | 4.8 | Data systems and processes | Is there a national system to monitor adverse events following immunisation (AEFIs)? | 6 | 6.79 | 8.97 | 4.62 |
| 5 | 3.1 | Data quality | Proportion of live births registered | 6 | 8.05 | 8.92 | 7.18 |
| 6 | 1.1 | Immunisation coverage | Number of districts with measles (MCV1) coverage in each range: <50%, 50-79%, 80-89%, 90-94, ≥95% | 6 | 7.35 | 8.29 | 6.41 |
| 7 | 1.1 | Immunisation coverage | Number of zero dose children, i.e. those that lack access to or are never reached by routine immunisation services (operationally measured as those who lack first dose of a DTP-containing vaccine) | 5 | 7.31 | 8.47 | 6.15 |
| 8 | 5.4 | VPD surveillance systems | Proportion of polio, measles, meningococcal disease, yellow fever, cholera, and Ebola outbreaks with timely detection and response | 4 | 6.34 | 8.84 | 3.85 |
| 9 | 3.5 | Data quality | Proportion of facility-level routine immunisation microplans with updated catchment area maps and strategy to reach them | 4 | 4.69 | 7.34 | 2.05 |
| 10 | 4.4 | Data systems and processes | Linkage of home-based records with civil birth registration through immunisation services | 3 | 5.01 | 7.45 | 2.56 |
| **Alternative ranking method 2: Ordered by 1) “keep” score, 2) weighted relevance score, 3) feasibility score** | | | | | | | |
| 1 | 2.1 | Use of insights | Country uses quality data on under-vaccinated to inform plans at community, subnational and national levels | 10 | 6.96 | 9.05 | 4.87 |
| 2 | 4.2 | Data systems and processes | Availability of sustainable and effective immunisation information system integrated within a robust national health information system | 6 | 6.43 | 9.01 | 3.85 |
| 3 | 1.11 | Immunisation coverage | Number of districts with measles (MCV2) coverage in each range: <50%, 50-79%, 80-89%, 90-94, ≥95% | 6 | 7.18 | 8.98 | 5.38 |
| 4 | 4.8 | Data systems and processes | Is there a national system to monitor adverse events following immunisation (AEFIs)? | 6 | 6.79 | 8.97 | 4.62 |
| 5 | 3.1 | Data quality | Proportion of live births registered | 6 | 8.05 | 8.92 | 7.18 |
| 6 | 5.4 | VPD surveillance systems | Proportion of polio, measles, meningococcal disease, yellow fever, cholera, and Ebola outbreaks with timely detection and response | 4 | 6.34 | 8.84 | 3.85 |
| 7 | 1.1 | Immunisation coverage | Number of zero dose children, i.e. those that lack access to or are never reached by routine immunisation services (operationally measured as those who lack first dose of a DTP-containing vaccine) | 5 | 7.31 | 8.47 | 6.15 |
| 8 | 5.1 | VPD surveillance systems | Non-polio acute flaccid paralysis (AFP) rate (target >1/100,000 among <15 years population) in a 12-month period | 2 | 6.36 | 8.36 | 4.36 |
| 9 | 1.1 | Immunisation coverage | Number of districts with measles (MCV1) coverage in each range: <50%, 50-79%, 80-89%, 90-94, ≥95% | 6 | 7.35 | 8.29 | 6.41 |
| 10 | 4.4 | Data systems and processes | Linkage of home-based records with civil birth registration through immunisation services | 3 | 5.01 | 7.45 | 2.56 |
| **Alternative ranking method 3: Ordered by 1) preference score, 2) crude relevance score, 3) feasibility score** | | | | | | | |
| 1 | 2.1 | Use of insights | Country uses quality data on under-vaccinated to inform plans at community, subnational and national levels | 10 | 6.96 | 9.05 | 4.87 |
| 2 | 1.11 | Immunisation coverage | Number of districts with measles (MCV2) coverage in each range: <50%, 50-79%, 80-89%, 90-94, ≥95% | 6 | 7.18 | 8.98 | 5.38 |
| 3 | 4.2 | Data systems and processes | Availability of sustainable and effective immunisation information system integrated within a robust national health information system | 6 | 6.43 | 9.01 | 3.85 |
| 4 | 3.1 | Data quality | Proportion of live births registered | 6 | 8.05 | 8.92 | 7.18 |
| 5 | 1.1 | Immunisation coverage | Number of districts with measles (MCV1) coverage in each range: <50%, 50-79%, 80-89%, 90-94, ≥95% | 6 | 7.35 | 8.29 | 6.41 |
| 6 | 4.8 | Data systems and processes | Is there a national system to monitor adverse events following immunisation (AEFIs)? | 6 | 6.79 | 8.97 | 4.62 |
| 7 | 1.1 | Immunisation coverage | Number of zero dose children, i.e. those that lack access to or are never reached by routine immunisation services (operationally measured as those who lack first dose of a DTP-containing vaccine) | 5 | 7.31 | 8.47 | 6.15 |
| 8 | 5.4 | VPD surveillance systems | Proportion of polio, measles, meningococcal disease, yellow fever, cholera, and Ebola outbreaks with timely detection and response | 4 | 6.34 | 8.84 | 3.85 |
| 9 | 3.5 | Data quality | Proportion of facility-level routine immunisation microplans with updated catchment area maps and strategy to reach them | 4 | 4.69 | 7.34 | 2.05 |
| 10 | 4.4 | Data systems and processes | Linkage of home-based records with civil birth registration through immunisation services | 3 | 5.01 | 7.45 | 2.56 |
| **Alternative ranking method 4: Ordered by 1) weighted relevance score, 2) feasibility score** | | | | | | | |
| 1 | 2.1 | Use of insights | Country uses quality data on under-vaccinated to inform plans at community, subnational and national levels | 10 | 6.96 | 9.05 | 4.87 |
| 2 | 4.2 | Data systems and processes | Availability of sustainable and effective immunisation information system integrated within a robust national health information system | 6 | 6.43 | 9.01 | 3.85 |
| 3 | 1.11 | Immunisation coverage | Number of districts with measles (MCV2) coverage in each range: <50%, 50-79%, 80-89%, 90-94, ≥95% | 6 | 7.18 | 8.98 | 5.38 |
| 4 | 4.8 | Data systems and processes | Is there a national system to monitor adverse events following immunisation (AEFIs)? | 6 | 6.79 | 8.97 | 4.62 |
| 5 | 3.1 | Data quality | Proportion of live births registered | 6 | 8.05 | 8.92 | 7.18 |
| 6 | 5.4 | VPD surveillance systems | Proportion of polio, measles, meningococcal disease, yellow fever, cholera, and Ebola outbreaks with timely detection and response | 4 | 6.34 | 8.84 | 3.85 |
| 7 | 4.6 | Data systems and processes | Proportion of districts having electronic vaccine and supply stock management system to monitor vaccine stock down to service delivery | 2 | 5.45 | 8.84 | 2.05 |
| 8 | 4.1 | Data systems and processes | Proportion of districts reporting stock availability (vaccines and supplies) at a service delivery level | 0 | 6.58 | 8.80 | 4.36 |
| 9 | 1.3 | Immunisation coverage | Dropout rates between first dose (DTP1) and first dose of measles-containing vaccine (MCV1) | 1 | 6.81 | 8.75 | 4.87 |
| 10 | 5.5 | VPD surveillance systems | Annual number of laboratory-confirmed epidemic-prone vaccine-preventable disease outbreaks | -1 | 6.27 | 8.70 | 3.85 |
| **Alternative ranking method 5: Ordered by 1) feasibility score, 2) weighted relevance score** | | | | | | | |
| 1 | 3.1 | Data quality | Proportion of live births registered | 6 | 8.05 | 8.92 | 7.18 |
| 2 | 1.1 | Immunisation coverage | Number of districts with measles (MCV1) coverage in each range: <50%, 50-79%, 80-89%, 90-94, ≥95% | 6 | 7.35 | 8.29 | 6.41 |
| 3 | 1.1 | Immunisation coverage | Number of zero dose children, i.e. those that lack access to or are never reached by routine immunisation services (operationally measured as those who lack first dose of a DTP-containing vaccine) | 5 | 7.31 | 8.47 | 6.15 |
| 4 | 3.2a | Data quality | Proportion of districts with complete and timely reporting from all health facilities | 2 | 6.93 | 7.70 | 6.15 |
| 5 | 1.2 | Immunisation coverage | Dropout rates between first dose (DTP1) and third dose (DPT3) of DTP-containing vaccine | 2 | 7.13 | 8.36 | 5.90 |
| 6 | 1.8 | Immunisation coverage | Number of districts with DTP3 coverage in each range: <50%, 50-79%, 80-89%, 90-94, ≥95% | 0 | 6.96 | 8.03 | 5.90 |
| 7 | 3.2 | Data quality | Proportion of districts with complete and timely reporting | -1 | 6.72 | 7.79 | 5.64 |
| 8 | 1.11 | Immunisation coverage | Number of districts with measles (MCV2) coverage in each range: <50%, 50-79%, 80-89%, 90-94, ≥95% | 6 | 7.18 | 8.98 | 5.38 |
| 9 | 3.6 | Data quality | Are the number of type-specific vaccine doses reported by age group (e.g. number of diphtheria cases by age group) based on recall, documentation, or both? | -1 | 6.68 | 8.24 | 5.13 |
| 10 | 2.1 | Use of insights | Country uses quality data on under-vaccinated to inform plans at community, subnational and national levels | 10 | 6.96 | 9.05 | 4.87 |

AEFI: Adverse events following immunisation; AFP: Acute flaccid paralysis; DTP: diphtheria-tetanus-pertussis; MCV: measles-containing vaccine; VPD: vaccine-preventable disease
