# Supplementary material for: Measuring what matters: Context-specific indicators for assessing immunisation performance in Pacific Island Countries and Areas
Source: PLOS Glob Public Health. 2024 Jul 25;4(7):e0003068. doi: 10.1371/journal.pgph.0003068 (PMC11271932; doi:10.1371/journal.pgph.0003068)
Supplement: S8 Appendix — (DOCX) [file pgph.0003068.s009.docx]

**Measuring what matters: context-specific indicators for assessing immunisation performance in Pacific Island Countries and Areas**

# S8 Appendix: Ten highest ranked indicators, by demographic and professional groups

| **Rank** | **Indicator ID** | **Category** | **Definition** | **Preference score** | **Mean feasibility-relevance score** | **Mean weighted relevance score** | **Mean feasibility score** |
| --- | --- | --- | --- | --- | --- | --- | --- |
| **All experts** | | | | | | | |
| 1 | 2.1 | Use of insights | Country uses quality data on under-vaccinated to inform plans at community, subnational and national levels | 10 | 6.96 | 9.05 | 4.87 |
| 2 | 3.1 | Data quality | Proportion of live births registered | 6 | 8.05 | 8.92 | 7.18 |
| 3 | 1.10 | Immunisation coverage | Number of districts with measles (MCV1) coverage in each range: <50%, 50-79%, 80-89%, 90-94, ≥95% | 6 | 7.35 | 8.29 | 6.41 |
| 4 | 1.11 | Immunisation coverage | Number of districts with measles (MCV2) coverage in each range: <50%, 50-79%, 80-89%, 90-94, ≥95% | 6 | 7.18 | 8.98 | 5.38 |
| 5 | 4.8 | Data systems and processes | Is there a national system to monitor adverse events following immunisation (AEFIs)? | 6 | 6.79 | 8.97 | 4.62 |
| 6 | 4.2 | Data systems and processes | Availability of sustainable and effective immunisation information system integrated within a robust national health information system | 6 | 6.43 | 9.01 | 3.85 |
| 7 | 1.1 | Immunisation coverage | Number of zero dose children, i.e. those that lack access to or are never reached by routine immunisation services (operationally measured as those who lack first dose of a DTP-containing vaccine) | 5 | 7.31 | 8.47 | 6.15 |
| 8 | 5.4 | VPD surveillance systems | Proportion of polio, measles, meningococcal disease, yellow fever, cholera, and Ebola outbreaks with timely detection and response | 4 | 6.34 | 8.84 | 3.85 |
| 9 | 3.5 | Data quality | Proportion of facility-level routine immunisation microplans with updated catchment area maps and strategy to reach them | 4 | 4.69 | 7.34 | 2.05 |
| 10 | 4.4 | Data systems and processes | Linkage of home-based records with civil birth registration through immunisation services | 3 | 5.01 | 7.45 | 2.56 |
| **Females** | | | | | | | |
| 1 | 1.10 | Immunisation coverage | Number of districts with measles (MCV1) coverage in each range: <50%, 50-79%, 80-89%, 90-94, ≥95% | 5 | 8.22 | 9.78 | 6.67 |
| 2 | 3.1 | Data quality | Proportion of live births registered | 4 | 8.11 | 9.08 | 7.14 |
| 3 | 1.11 | Immunisation coverage | Number of districts with measles (MCV2) coverage in each range: <50%, 50-79%, 80-89%, 90-94, ≥95% | 4 | 7.65 | 9.11 | 6.19 |
| 4 | 5.4 | VPD surveillance systems | Proportion of polio, measles, meningococcal disease, yellow fever, cholera, and Ebola outbreaks with timely detection and response | 4 | 6.46 | 8.17 | 4.76 |
| 5 | 2.1 | Use of insights | Country uses quality data on under-vaccinated to inform plans at community, subnational and national levels | 4 | 6.08 | 8.36 | 3.81 |
| 6 | 4.8 | Data systems and processes | Is there a national system to monitor adverse events following immunisation (AEFIs)? | 3 | 6.91 | 9.06 | 4.76 |
| 7 | 4.4 | Data systems and processes | Linkage of home-based records with civil birth registration through immunisation services | 3 | 5.52 | 7.71 | 3.33 |
| 8 | 3.5 | Data quality | Proportion of facility-level routine immunisation microplans with updated catchment area maps and strategy to reach them | 3 | 5.13 | 7.40 | 2.86 |
| 9 | 4.2 | Data systems and processes | Availability of sustainable and effective immunisation information system integrated within a robust national health information system | 3 | 4.82 | 8.22 | 1.43 |
| 10 | 1.1 | Immunisation coverage | Number of zero dose children, i.e. those that lack access to or are never reached by routine immunisation services (operationally measured as those who lack first dose of a DTP-containing vaccine) | 2 | 8.29 | 10.39 | 6.19 |
| **Males** | | | | | | | |
| 1 | 2.1 | Use of insights | Country uses quality data on under-vaccinated to inform plans at community, subnational and national levels | 6 | 7.98 | 9.85 | 6.11 |
| 2 | 4.2 | Data systems and processes | Availability of sustainable and effective immunisation information system integrated within a robust national health information system | 3 | 8.30 | 9.93 | 6.67 |
| 3 | 4.8 | Data systems and processes | Is there a national system to monitor adverse events following immunisation (AEFIs)? | 3 | 6.66 | 8.87 | 4.44 |
| 4 | 1.1 | Immunisation coverage | Number of zero dose children, i.e. those that lack access to or are never reached by routine immunisation services (operationally measured as those who lack first dose of a DTP-containing vaccine) | 3 | 6.33 | 6.55 | 6.11 |
| 5 | 3.1 | Data quality | Proportion of live births registered | 2 | 8.00 | 8.77 | 7.22 |
| 6 | 3.8 | Data quality | Proportion of districts reporting at least 90% on time during a one-year period for suspected cases for all priority vaccine-preventable diseases under nationwide surveillance, including reporting of zero cases | 2 | 7.46 | 8.81 | 6.11 |
| 7 | 3.2a | Data quality | Proportion of districts with complete and timely reporting from all health facilities | 2 | 7.01 | 6.79 | 7.22 |
| 8 | 5.1 | VPD surveillance systems | Non-polio acute flaccid paralysis (AFP) rate (target >1/100,000 among <15 years population) in a 12-month period | 2 | 6.97 | 10.06 | 3.89 |
| 9 | 1.2 | Immunisation coverage | Dropout rates between first dose (DTP1) and third dose (DPT3) of DTP-containing vaccine | 2 | 6.71 | 6.75 | 6.67 |
| 10 | 1.11 | Immunisation coverage | Number of districts with measles (MCV2) coverage in each range: <50%, 50-79%, 80-89%, 90-94, ≥95% | 2 | 6.65 | 8.85 | 4.44 |
| **Immunisation and public health experts** | | | | | | | |
| 1 | 2.1 | Use of insights | Country uses quality data on under-vaccinated to inform plans at community, subnational and national levels | 7 | 7.41 | 9.48 | 5.33 |
| 2 | 1.11 | Immunisation coverage | Number of districts with measles (MCV2) coverage in each range: <50%, 50-79%, 80-89%, 90-94, ≥95% | 6 | 7.35 | 9.70 | 5.00 |
| 3 | 4.8 | Data systems and processes | Is there a national system to monitor adverse events following immunisation (AEFIs)? | 5 | 7.80 | 10.60 | 5.00 |
| 4 | 1.10 | Immunisation coverage | Number of districts with measles (MCV1) coverage in each range: <50%, 50-79%, 80-89%, 90-94, ≥95% | 5 | 7.60 | 8.87 | 6.33 |
| 5 | 1.1 | Immunisation coverage | Number of zero dose children, i.e. those that lack access to or are never reached by routine immunisation services (operationally measured as those who lack first dose of a DTP-containing vaccine) | 5 | 7.56 | 9.11 | 6.00 |
| 6 | 4.2 | Data systems and processes | Availability of sustainable and effective immunisation information system integrated within a robust national health information system | 5 | 6.94 | 9.87 | 4.00 |
| 7 | 3.1 | Data quality | Proportion of live births registered | 4 | 8.32 | 9.97 | 6.67 |
| 8 | 5.1 | VPD surveillance systems | Non-polio acute flaccid paralysis (AFP) rate (target >1/100,000 among <15 years population) in a 12-month period | 4 | 7.69 | 9.71 | 5.67 |
| 9 | 3.5 | Data quality | Proportion of facility-level routine immunisation microplans with updated catchment area maps and strategy to reach them | 4 | 5.75 | 8.82 | 2.67 |
| 10 | 4.4 | Data systems and processes | Linkage of home-based records with civil birth registration through immunisation services | 3 | 5.72 | 8.78 | 2.67 |
| **Health information system experts** | | | | | | | |
| 1 | 2.1 | Use of insights | Country uses quality data on under-vaccinated to inform plans at community, subnational and national levels | 3 | 5.48 | 7.62 | 3.33 |
| 2 | 3.1 | Data quality | Proportion of live births registered | 2 | 7.33 | 5.77 | 8.89 |
| 3 | 1.2 | Immunisation coverage | Dropout rates between first dose (DTP1) and third dose (DPT3) of DTP-containing vaccine | 2 | 6.57 | 7.59 | 5.56 |
| 4 | 5.4 | VPD surveillance systems | Proportion of polio, measles, meningococcal disease, yellow fever, cholera, and Ebola outbreaks with timely detection and response | 2 | 2.94 | 4.76 | 1.11 |
| 5 | 3.2a | Data quality | Proportion of districts with complete and timely reporting from all health facilities | 1 | 6.72 | 5.66 | 7.78 |
| 6 | 3.2 | Data quality | Proportion of districts with complete and timely reporting | 1 | 6.57 | 6.48 | 6.67 |
| 7 | 1.10 | Immunisation coverage | Number of districts with measles (MCV1) coverage in each range: <50%, 50-79%, 80-89%, 90-94, ≥95% | 1 | 6.03 | 5.39 | 6.67 |
| 8 | 5.5 | VPD surveillance systems | Annual number of laboratory-confirmed epidemic-prone vaccine-preventable disease outbreaks | 1 | 5.00 | 5.56 | 4.44 |
| 9 | 4.2 | Data systems and processes | Availability of sustainable and effective immunisation information system integrated within a robust national health information system | 1 | 4.73 | 6.14 | 3.33 |
| 10 | 1.3 | Immunisation coverage | Dropout rates between first dose (DTP1) and first dose of measles-containing vaccine (MCV1) | 1 | 4.69 | 6.05 | 3.33 |
| **Experts working in one Pacific Island Country and Territory** | | | | | | | |
| 1 | 4.8 | Data systems and processes | Is there a national system to monitor adverse events following immunisation (AEFIs)? | 6 | 7.49 | 9.43 | 5.56 |
| 2 | 2.1 | Use of insights | Country uses quality data on under-vaccinated to inform plans at community, subnational and national levels | 5 | 6.90 | 8.81 | 5.00 |
| 3 | 1.11 | Immunisation coverage | Number of districts with measles (MCV2) coverage in each range: <50%, 50-79%, 80-89%, 90-94, ≥95% | 4 | 8.02 | 9.37 | 6.67 |
| 4 | 4.2 | Data systems and processes | Availability of sustainable and effective immunisation information system integrated within a robust national health information system | 3 | 8.01 | 10.47 | 5.56 |
| 5 | 3.3 | Data quality | Proportion of districts reporting negative DTP1-DTP3 drop out | 3 | 6.05 | 7.09 | 5.00 |
| 6 | 3.1 | Data quality | Proportion of live births registered | 2 | 8.60 | 8.86 | 8.33 |
| 7 | 5.3 | VPD surveillance systems | Access to laboratory capacity to test for at least one bacterial vaccine-preventable disease (VPD) | 2 | 7.90 | 10.79 | 5.00 |
| 8 | 3.2a | Data quality | Proportion of districts with complete and timely reporting from all health facilities | 2 | 7.51 | 7.79 | 7.22 |
| 9 | 1.10 | Immunisation coverage | Number of districts with measles (MCV1) coverage in each range: <50%, 50-79%, 80-89%, 90-94, ≥95% | 2 | 7.21 | 7.76 | 6.67 |
| 10 | 5.5 | VPD surveillance systems | Annual number of laboratory-confirmed epidemic-prone vaccine-preventable disease outbreaks | 2 | 6.91 | 9.93 | 3.89 |
| **Experts working in more than one Pacific Island Country and Territory** | | | | | | | |
| 1 | 2.1 | Use of insights | Country uses quality data on under-vaccinated to inform plans at community, subnational and national levels | 5 | 7.01 | 9.26 | 4.76 |
| 2 | 1.1 | Immunisation coverage | Number of zero dose children, i.e. those that lack access to or are never reached by routine immunisation services (operationally measured as those who lack first dose of a DTP-containing vaccine) | 4 | 8.29 | 10.39 | 6.19 |
| 3 | 3.1 | Data quality | Proportion of live births registered | 4 | 7.58 | 8.97 | 6.19 |
| 4 | 1.10 | Immunisation coverage | Number of districts with measles (MCV1) coverage in each range: <50%, 50-79%, 80-89%, 90-94, ≥95% | 4 | 7.50 | 8.81 | 6.19 |
| 5 | 1.8 | Immunisation coverage | Number of districts with DTP3 coverage in each range: <50%, 50-79%, 80-89%, 90-94, ≥95% | 3 | 7.39 | 9.55 | 5.24 |
| 6 | 5.4 | VPD surveillance systems | Proportion of polio, measles, meningococcal disease, yellow fever, cholera, and Ebola outbreaks with timely detection and response | 3 | 5.58 | 7.83 | 3.33 |
| 7 | 4.2 | Data systems and processes | Availability of sustainable and effective immunisation information system integrated within a robust national health information system | 3 | 5.07 | 7.76 | 2.38 |
| 8 | 4.6 | Data systems and processes | Proportion of districts having electronic vaccine and supply stock management system to monitor vaccine stock down to service delivery | 3 | 4.73 | 8.50 | 0.95 |
| 9 | 3.5 | Data quality | Proportion of facility-level routine immunisation microplans with updated catchment area maps and strategy to reach them | 3 | 4.66 | 7.90 | 1.43 |
| 10 | 1.2 | Immunisation coverage | Dropout rates between first dose (DTP1) and third dose (DPT3) of DTP-containing vaccine | 2 | 7.89 | 9.59 | 6.19 |
