# Supplementary material for: Measuring what matters: Context-specific indicators for assessing immunisation performance in Pacific Island Countries and Areas
Source: PLOS Glob Public Health. 2024 Jul 25;4(7):e0003068. doi: 10.1371/journal.pgph.0003068 (PMC11271932; doi:10.1371/journal.pgph.0003068)
Supplement: S9 Appendix — (DOCX) [file pgph.0003068.s010.docx]

**Measuring what matters: context-specific indicators for assessing immunisation performance in Pacific Island Countries and Areas**

# S9 Appendix: Relevance versus feasibility scores for indicators, by demographic and professional groups

Note: Relevance scores are the weighted relevance scores.

A. Females B. Males

C. Immunisation and public health experts D. Health information system experts

E. Experts working in one Pacific Island Country and Territory F. Experts working in more than one Pacific Island Country and Territory
