# Supplementary material for: Measuring what matters: Context-specific indicators for assessing immunisation performance in Pacific Island Countries and Areas
Source: PLOS Glob Public Health. 2024 Jul 25;4(7):e0003068. doi: 10.1371/journal.pgph.0003068 (PMC11271932; doi:10.1371/journal.pgph.0003068)
Supplement: Attachment — Submitted filename: Author responses to reviewers_final.docx [file pgph.0003068.s011.docx]

Measuring what matters: context-specific indicators for assessing immunisation performance in Pacific Island Countries and Areas

Response to reviewers’ comments

| **Editor or reviewer comment** | **Authors’ response** | **Changes to manuscript** |
| --- | --- | --- |
| **Editor’s comments** |  |  |
| Please review your reference list to ensure that it is complete and correct. If you have cited papers that have been retracted, please include the rationale for doing so in the manuscript text, or remove these references and replace them with relevant current references. Any changes to the reference list should be mentioned in the rebuttal letter that accompanies your revised manuscript. If you need to cite a retracted article, indicate the article’s retracted status in the References list and also include a citation and full reference for the retraction notice. | We thank the Editor and all reviewers for their thoughtful comments and have given them all our full attention.  We have reviewed our reference list when revising our manuscript, and confirm that none of the papers cited have been retracted.  We have made minor amendments to the reference list due to adding references for the analysis software used. | Addition of two references |
| Please include a complete copy of PLOS’ questionnaire on inclusivity in global research in your revised manuscript. Our policy for research in this area aims to improve transparency in the reporting of research performed outside of researchers’ own country or community. The policy applies to researchers who have travelled to a different country to conduct research, research with Indigenous populations or their lands, and research on cultural artefacts. The questionnaire can also be requested at the journal’s discretion for any other submissions, even if these conditions are not met. Please find more information on the policy and a link to download a blank copy of the questionnaire here: https://journals.plos.org/globalpublichealth/s/best-practices-in-research-reporting. Please upload a completed version of your questionnaire as Supporting Information when you resubmit your manuscript. | Thank you for this comment, we had uploaded a copy of the questionnaire on inclusivity in global health research with our original submission and have included it with the revised manuscript. | No changes to manuscript; questionnaire on inclusivity in global health attached to submission |
| We have noticed that you have uploaded Supporting Information files, but you have not included a list of legends. Please add a full list of legends for your Supporting Information files after the references list. | We have now included a list of legends for our file with Supporting Information in the manuscript after references as requested. | List of legends for Supporting Information included after references |
| **Reviewer #1** |  |  |
| Summary: This paper outlines findings from a series of expert consultations conducted with the objective to provide insight on the utility of various immunisation system performance indicators for decision-making. The study authors should be commended for their interesting research question and appreciation of variation in needs across Pacific Island countries (PICs). This paper, however, could benefit from a more thorough discussion of the specific types of decisions that these personnel are making across their respective settings and responsibilities and how these types of decisions may influence their feedback on the suggested indicators. | Thank you for these thoughtful comments.  Based on reviewer feedback, we have included some more information in the introduction section to provide some pertinent background on immunisation systems and decision-making for immunisation in PICs. The related text is shown in the next column.  We have further added some text to the manuscript in the findings section. This is described in response to the reviewer’s related comment below. | Addition of paragraph prior to statement of aims in the introduction: pages 4-5, lines 95–111:  Added text: With regards to decision-making for immunisation, PICs independently evaluate and make decisions about their immunisation programs, including decisions about how immunisation services are delivered (including catch-up campaigns), new vaccine introductions, and allocation of resources. Decisions are made by staff at the country’s respective ministry or department of health, with some countries having advisory and decision-making committees. No PIC has formally established a national immunisation technical advisory group due to resource constraints, and some immunisation actors contribute to decision-making both within their country and across the region. Most PICs (i.e. other than those aligned with USA or France) obtain vaccines from the Vaccine Independence Initiative with support for procurement from UNICEF.^11^ Development partners (especially the World Health Organization and UNICEF with more limited support from the US Centers for Disease Prevention and Control) provide technical support in various areas including program planning, cold chain and waste management, vaccine safety and disease surveillance, both at the country-level and throughout the region. Only a handful of countries (namely Kiribati and the Solomon Islands) receive Gavi support, with financial contributions largely through vaccine procurement and technical assistance from various nongovernmental and bilateral donors. Within this complex setting, we aimed to identify which indicators are critical to measure immunisation performance in PICs and the reasons for selecting these indicators. |
| Major comments:  1. Useful indicators across any setting and participant (given their affiliation) are likely to differ based on the underlying decision that is being made. Can you speak to the variation in decisions that are being considered across PICs (e.g., planning tailored campaigns by geographically or age, considerations for new vaccine introduction, etc.) and how this influences the relevance of the various indicators considered? This is likely the largest underlying driver of responses and was barely touched on. | Thank you for this comment. We have discussed some notable differences in preferences in for indicators based on experts’ roles (i.e. immunisation/public health expert vs health information specialist) and their geographical scope of work (i.e. national vs regional) in the results on page 21, paragraph beginning on line 325, as well as how experts’ individual and organisational priorities affected their preferences for indicators under the heading “C. Priorities and experiences of different immunisation actors”, page 28, lines 498-512.  We have briefly discussed that experts selected indicators that inform considerations related to programmatic decision-making (section on usefulness for decision-making and planning, starting page 22, line 341), but agree that we can enhance this section with some examples. We have added these examples in the text. | Text added to results section, “1. Usefulness or decision-making and planning”.  Examples on how program planners use data, page 22, lines 355-364: Experts involved in planning immunisation programs highlighted the importance of having data on stock availability and management at all levels of the health system to ensure immunisation services aren’t interrupted, to take action to understand the cause of stockouts and resolve them promptly, and to undertake planning to minimise stockouts in the future. Additionally, experts highlighted the need to know where populations, particularly undervaccinated children, are to develop microplans for service delivery and target resources appropriately:  *“This one was important, more so than the other indicators, because it shows you know where the unvaccinated children are, and what we need to do to go and reach them, what resources you need, how many staff you need, how much diesel you need, how many boats you need to actually access those communities.” (Immunisation expert working across multiple PICS)*  Page 23, lines 375-378: Experts involved in planning programs indicated how low measles coverage was a trigger for them to decide whether supplementary immunisation activities were required.  Example related to system-level initiative, page 23, line 383-386: In another example, experts discussed how reporting of adverse events following immunisation was known to be problematic in their setting, and so they prioritised indicators related to examining if the safety surveillance system was established and if events were being reported. |
| 2. To increase transparency, as well as provide more robust context for the expertise provided, study authors could provide the names, countries and affiliations of the experts consulted per this investigation somewhere in the report (e.g., as co-authors, acknowledgements, in the supplement). | Thank you for this comment. We agree on the need for transparency in research, but we are unfortunately unable to provide a detailed list of the names, countries and affiliations of experts who participated in this study. Our ethical obligations require us to ensure the confidentiality of study participants. As part of the consent process, we informed study participants that their names and other personal identifying information will remain confidential, and that we will not include identifying information in any published materials. We have instead included a summary of participant characteristics in our study findings, and mention the countries represented in our study in the findings section. We are unable to provide more detailed or individual-level characteristics as the health workforce in Pacific Island Countries is small and it is likely that any further details will enable identification of participants by those who know them.  If the reviewer requires further information, they are able to approach the ANU Research Ethics Committee ([human.ethics.officer@anu.edu.au](mailto:human.ethics.officer@anu.edu.au)) via the PLOS Editorial team, referencing protocol 2022/368. | No changes to manuscript |
| a. Also, study authors should consider adding a section or information on how many participants were from countries with populations < 100,000 or some other metric. | While we can add such a statistic, we are concerned this will be misleading. Seven of the 13 experts in our study worked across more than one country in the region, with some from development partner organisations working across most countries in the region. Their perspectives would therefore cover their experiences in PICs with both smaller and larger population sizes. As we are unable to conduct an analysis based on such a metric (as some experts would have worked in countries both with >100,000 and with <100,000 people, resulting in double-counting), we have not reported this in our manuscript. We also note that sharing these details may make some individuals identifiable to those who know them, given we have named specific countries represented by our included experts.  While we prefer not to share these details publicly, we are happy to share some further details about our expert participants in confidence. Our expert participants included:   - 7 participants who worked across more than one PIC, of whom 4 indicated they worked across the region including in countries with populations <100,000 people - 6 participants who worked at the national level only, of whom 1 worked in a country with population <100,000 people.   We would like to add that these numbers should be taken as a minimum indication of how many of our experts worked in countries with populations <100,000 people; some experts may also have contributed to decision-making in other countries based on their capacity as experts on advisory committees. The numbers above are based on the information expert participants provided to the research team.  To provide readers with some context, we have alluded to the fact that immunisation actors in the Pacific work both in a national context as well as across the region in text that we have added to the introduction section. | Text added to introduction section for context, page 5, lines 99-107:  Added text: No PIC has formally established a national immunisation technical advisory group due to resource constraints, and some immunisation actors contribute to decision-making both within their country and across the region. Most PICs (i.e. other than those aligned with USA or France) obtain vaccines from the Vaccine Independence Initiative with support for procurement from UNICEF.^11^ Development partners (especially the World Health Organization and UNICEF with more limited support from the US Centers for Disease Prevention and Control) provide technical support in various areas including program planning, cold chain and waste management, vaccine safety and disease surveillance, both at the country-level and throughout the region. |
| b. Are countries with now coverage uniformly nationally versus those with substantial variation more likely to prefer certain indicators? Study authors mention this theme starting on line 450, but it feels incomplete and non-substantive. | Thank you for this comment, we agree that differences in preferences for indicators based on variation, or lack thereof, in immunisation coverage is an important point. As more than half our experts worked across multiple countries, it would be challenging to examine this in the quantitative results. However, this issue did arise in the qualitative discussions. We have included a description of how current levels of coverage of childhood immunisations affected preferences for certain indicators, particularly focusing on zero-dose coverage, in the section on how differences in health priorities affected indicator preferences (see section “A. Differences in health priorities” starting on page 26 line 450, particularly lines 452-460 where we discuss that zero-dose vaccination was considered to be a bigger problem in countries with lower coverage of routine childhood vaccinations). We agree that this discussion can be enhanced, and have added another example to the text.  In addition, we describe how preferences for indicators were influenced by individuals’ scope of work (i.e. some were more interested in indicators that they were accountable for) in the paragraph starting at line 507.  We have also added a line regarding the irrelevance of subnational denominations for countries with very small population sizes. | Page 27, line 464:  Added text: Another expert raised that closely tracking coverage of booster doses of DTP vaccine would be more influential in strengthening their program, as coverage of the fourth, fifth and sixth doses of DTP vaccine were known to be low whereas coverage of DTP vaccine doses given in infancy is high.  Page 29, line 539-540:  Experts also noted that subnational denominations were irrelevant in PICs where the population sizes are very small and health services are highly centralised. |
| c. Study authors illude to some of this information possibly being contained within the appendix (line 294)., but it is buried among a lot of information and needs to be more clearly highlighted. | We have highlighted some of the main differences in indicator preferences between groups in the paragraph highlighted (starting on line 325), but agree this can be enhanced. We have added some further text to this section in the results. | Page 21, lines 329–335, text edited and additional line added:  Monitoring the establishment of adverse events surveillance was the highest ranked indicator among nationally-based experts, but ranked 20^th^ among experts working in more than one PIC. Both groups ranked the use of data on under-vaccinated to inform planning and measles vaccination coverage amongst the top ten of all 41 indicators. Health information specialists ranked indicators related to the timeliness and completeness of immunisation data higher than immunisation specialists (5^th^ and 6^th^ versus 15^th^ and 27^th^), albeit noting the small number of health information specialists in the study limits interpretation of this finding. |
| 3. Without adding information on which indicator is which to each point in figures 2-3, they aren’t actually very informative or interesting to the reader. At minimum, study authors could colour the points instead by indicator category instead of top/middle/bottom thirds (which is easy to see visually without colour indication). | Thank you for this suggestion. We have updated the graphs to indicate the indicator category by colour and top/middle/bottom thirds by shape. We generated a similar graph that includes labels for each indicator but the overlapping labels makes it difficult to read, so we have not included this. We can provide this if requested. | Figures 2 and 3 updated |
| Minor comments:  1. Line 56. Perhaps give a few examples of “indicators” to paint a clearer image for the reader. Something like “These immunisation-based indicators include more traditional metrics such as MCV1 coverage and DTP1-3 dropout and other less common metrics such as XXXX.” | Thank you, we have made the changes suggested. | Page 3, lines 56-60  Line added: This included widely-used routine indicators on vaccination coverage (e.g. coverage of the first dose of measles vaccine) and lesser-known indicators measuring performance of various aspects of the immunisation system (e.g. proportion of districts with electronic vaccine and supply stock management systems to monitor vaccine stock). |
| 2. Lines 79-83. Can you say anything yet about the quality of data from these electronic immunisation registers? Given many underlying biases of vaccination coverage administrative data, are these systems likely experiencing similar (or different) challenges regarding representativeness, completeness, etc? | Thank you for raising this, we agree that in general there is variation in the quality of data from electronic immunisation registers. Unfortunately we are unaware of any documentation (peer-reviewed or grey literature) on the quality of data from EIRs used in PICs. In fact, other than in the US Affiliated Pacific Island (USAPI) territories, no functional registers exist. Even with those in the USAPI, there are well known data quality issues (personal communication with data custodians, MS). Our intention in this section was to provide some background on the variation in data systems for immunisation across PICs and maturity of digital information systems, and hope that omission of further detail on the quality of data from these systems does not impact this. We have instead added some text to indicate the usefulness of having these systems.  For the peer-reviewer’s reference, the study author team is also undertaking a study to examine the role of electronic immunisation registers in increasing data use in decision-making for immunisation in Vanuatu, which includes a component assessing data quality. We will report this results in a forthcoming manuscript. | Page 4, lines 85-86:  Text added: …which have enabled more varied analysis of immunisation indicators and rapid assessments of coverage among at-risk populations during outbreaks. |
| 3. The term “stakeholder” is used multiple times throughout the manuscript. Study authors should reflect on the use of this term and how it might perpetuate colonialism within the broader global health field. Please consider a more mindful term, such as “affected parties” or “decision-makers”. See https://www.cdc.gov/healthcommunication/Preferred_Terms.html for additional examples. | Thank you for this comment. We have reviewed our use of the word “stakeholder” and replaced it with a more suitable term. Please note we have not changed the once instance where it is mentioned in a direct quote as changing this would be inappropriate. | Changes on the following pages:  Abstract, p. 2, line 42: changed to “immunisation actors”  Methods – study design, p. 6, line 127: changed to “immunisation actors”  Methods – recruitment and study population, p. 5 line 138: changed to “professionals” |
| 4. Are the methods for “preference” score validated? Or borrowed from other research? It would be helpful to know if study authors crafted this method themselves or if there is any validation of these methods? If independently derived, have study authors done any sensitivity analyses on methodologic choices? | We would like to clarify that we derived the methods for calculating the preference scores in this study. Other studies have elicited expert opinion of specific indicator characteristics in a similar manner to the way we have assessed feasibility and relevance (see references below). Other studies have also asked experts to rank indicators, but given the number of indicators (n=41), we considered asking experts to select their preferred indicators to be more feasible. Our knowledge of the literature (see references below) and experience of colleagues doing similar studies showed that mean scores for specific criteria are often very similar and are difficult to use to differentiate between what is truly important to measure compared with what is not. Mean scores alone also do not reflect the variation in experts’ individual scores, with indicators receiving a wide range of values often having similar mean values as those with a small range of scores. Given these limitations and the potential to miss factors other than feasibility and relevance affecting indicator selection, we included the question on preferences for indicators as a way to differentiate how important experts believed it was to measure indicators with similar mean scores for feasibility and relevance. We initially planned to use preferences to identify whether the vast majority of experts (i.e. 75% or more) agreed that a certain indicator should be included or excluded from a list of common indicators for all PICs to narrow the list of indicators for discussion. However our preliminary results showed substantial variation in preferences among experts, so we adapted our approach to calculate a preference score for each indicator and then used this to rank the order of indicators.  We tested different ways of calculating and using the preference scores, including ranking indicators based on the number of experts who said they would “keep” an indicator only (i.e. disregarding those who said they would “remove” the indicator), ranking first by the number who said they would “keep” the indicator followed by the number who said they would “remove” the indicator, and based on feasibility-relevance scores alone (i.e. disregarding the preference scores). While the different methods resulting in a slightly different order of the ranked indicators, the results overall were unchanged. Indeed, the ten highest ranked indicators remained almost identical regardless of the method of ranking used. We elected to go ahead with the method of calculating preference scores as described in the paper as it provided a way of accounting for both the number of people who wanted to “keep” the indicator and those who wanted to “remove” it, accounting for the variation in preferences for indicators.  Related references:   - Ahuja S, Gronholm PC, Shidhaye R, Jordans M, Thornicroft G. Development of mental health indicators at the district level in Madhya Pradesh, India: mixed methods study. BMC Health Serv Res. 2018;18(1):867. - Carinci F, Van Gool K, Mainz J, Veillard J, Pichora EC, Januel JM, et al. Towards actionable international comparisons of health system performance: expert revision of the OECD framework and quality indicators. International Journal for Quality in Health Care. 2015;27(2):137-146. - Grube MM, Scheidt-Nave C, Gaertner B, Lüdtke D, Bosnjak M, Heinrich S, et al. The selection and definition of indicators in public health monitoring for the 65+ age group in Germany. J Health Monit. 2019;4(2):86–101. - Jordans MJD, Chisholm D, Semrau M, Upadhaya N, Abdulmalik J, Ahuja S, et al. Indicators for routine monitoring of effective mental healthcare coverage in low- and middle-income settings: a Delphi study. Health Policy Plan. 2016;31(8):1100–6. - Rich R, D’Hont T, Murphy KE, Veillard J, Chatwood S. Selecting contextually appropriate performance indicators in a circumpolar context: a modified Delphi approach. BMC Health Serv Res. 2021;21(1):530. | We have added a line to the methods and findings sections, each, to indicate that we conducted a sensitivity analysis, and that using different ranking criteria did not change our study conclusions.  Text added, page 11, lines 229-231: We conducted a sensitivity analysis to examine the effect of using different rating criteria to rank indicators. Analyses were conducted in Stata 17, and graphics were produced using R.  Text added, page 21, lines 321-323: In our sensitivity analysis, the highest ranked indicators remained largely unchanged, with the most differences noted when indicators were ranked by feasibility scores first (see Appendix 7).  We have also added a section in the appendix showing the top ten ranked indicators based on different ranking methods for transparency. |
| a. On average, how many indicators did each expert choose to keep versus remove? This would give some more context to the quantitative preference scores that are otherwise difficult to interpret. | We asked experts to select a specific number of indicators to keep and remove from each indicator category, amounting to 25% of the indicators in each category:   - Immunisation coverage: 3 of 12 indicators - Use of insights: 1 of 4 indicators - Data quality: 2 of 9 indicators - data systems and processes for collecting and reporting immunisation data: 3 of 10 indicators - VPD surveillance system: 2 of 6 indicators   In total, we asked experts to select 11 indicators to keep and 11 indicators to remove out of a total of 41 indicators. | We have added the total number of indicators that experts were asked to keep or remove to Table 1, where we describe our rating criteria.  Page 9, Table 1 (added text in red font):  Experts were asked to select a specified number of indicators (i.e. top 25% and bottom 25%, i.e. total of 11 each) to “keep” and “remove” within each of the five categories |
| **Reviewer #2** |  |  |
| Thank you for the opportunity to review this manuscript which reports the findings of an expert elicitation study conducted to better understand how experts prioritise indicators of national immunisation performance in Pacific Island Countries and Areas. The outcomes of this study are quite interesting and should help inform global partners on how best to tailor indicators for settings with varying contexts. Overall, the manuscript is very well written, the methodology outlined is sound, and the implications drawn are relevant for the field. I have the following minor comments for the authors’ attention. | We thank the reviewer for their consideration of our work. | No changes required for this comment |
| Line 81, page 4; “During the COVID-19, some PICs introduced new digital systems…” Kindly insert “pandemic” after “COVID-19”. | Thank you for noting this typo, we have added the word “pandemic” as suggested. | Word “pandemic” added, line 88 |
| Line 82, page 4; “…to collect and aggregate immunisation data pandemic, and are at varying stages…” Kindly review this statement and rephrase appropriately for ease of clarity. | Thank you, we have attempted to clarify this sentence by rewording the latter part. | Page 4, line 87  Text edited: some PICs introduced new digital systems to collect and aggregate immunisation data pandemic, with progress in implementation varying across countries. |
| Lines 158 – 159, page 9; “…minor revisions, the elicitation instrument was finalised.” For transparency and reproducibility, kindly expand on what amendments were made to the instrument after the piloting process. | We have added some details on the types of changes made in this section. | Page 10, lines 180-182  Text added: Following minor revisions to improve the clarity of included questions and instructions to respondents, consistency in terminology and order of questions, the elicitation instrument was finalised. |
| Line 165, page 9; “Experts then independently completed the elicitation instrument…” The authors should indicate in what language(s) the instrument was administered and whether translation / back translation was required for the purpose of analysis. This should also be clarified with regards to the qualitative discussions. | Thank you for raising this. The study was conducted entirely in English. None of the potential participants that we contacted requested that the study materials be available in another language. We have included a sentence to indicate the study was conducted in English. | Page 11, line 207-208  Text added: The entire study, including instrument administration and discussions, was conducted in English only. |
| Line 188 – 189, page 10; “Informed consent was obtained verbally during the first meeting.” The authors should expand on this, indicating why written informed consent was not required/appropriate for a study of this design. | We obtained both verbal and written consent from experts. We obtained verbal agreement first before sending experts an individualised link to the online elicitation instrument. The first page of the elicitation instrument asked participants to confirm their agreement to participate, and required them to indicate “yes” or “no”. This process received ethical approval. We have attempted to clarify that we obtained written consent in the text. | Page 10, line 214:  Text added: Experts were asked to confirm their consent to participate in writing at the time they completed the elicitation instrument online. |
| Line 227, page 11; “Twenty-two experts agreed to participate in the study…” Kindly provide clarity on non-responders or participants who did not complete the study. Furthermore, based on their roles, representation, and characteristics, it will also be important to understand within the study limitations section whether those who did not respond after agreeing to participate or did not complete the study may have contributed any further significant findings to this study than what was captured from the 13 experts. | Thank you for raising this – we have added some text to describe the experts who initially agreed to participate but did not complete the study. We have added a line in the limitations to discuss this. Non-completion was most likely due to their workloads and lack of time rather than reluctance to participate, which two of the three who completed the first round of the elicitation but not the second confirmed with us in writing.  Our limitation section includes some discussion on selection and sampling bias, as we acknowledge that this may have introduced bias into the study (please see the paragraph beginning on page 33, line 634). | Page 13, lines 259-263  Text added: Among those who initiated (i.e. met with the research team and provided verbal agreement to participate or completed part of the study) but did not complete their participation in the study, five were affiliated with development partner organisations or research institutions, while three were country-level staff with their respective ministries of health (one immunisation expert and two health information specialists).  Page 36, line 670-672  Text added: Non-participation of some experts, including non-responders and experts who began but did not complete the study, meant that some countries may not have been adequately represented in this study. |
| Line 486, page 26; “…were asked about their preferences were for characteristics of a set of indicators…” This sentence is unclear and needs rephrasing. | Thank you, we have edited the text to clarify what we asked participants. | Page 29, lines 543-545  Text edited: we asked experts how many indicators they believe a single set of indicators to monitor immunisation performance nationally should be limited to. |
| Lines 492 – 493, page 27; “Experts said fewer insights to be more likely to be reviewed and used than long lists of insights…” Kindly rephrase this statement for ease of clarity. | Thank you, we have edited the text to clarify its meaning. | Page 28, lines 550-552  Text edited: Experts said fewer insights to be more likely to be reviewed and used by decision-makers in contrast to data on a large volume of indicators, and therefore lead to concrete actions |
| Line 594, page 32; “…may have provided greater opportunity build connections and clarify questions.” Kindly rephrase for clarity. | Thank you, we have edited the text to add more details and clarify this sentence. | Page 35, lines 654-655  Text edited: … whereas a face-to-face elicitation may have provided greater opportunity build rapport with experts and clarify experts’ questions about the study including specific aspects of the instrument. |
